# 2,3-*cis*-2R,3R-(−)-epiafzelechin-3-*O*-p-coumarate, a novel flavan-3-ol isolated from *Fallopia convolvulus* seed, is an estrogen receptor agonist in human cell lines

**DOI:** 10.1186/1472-6882-13-133

**Published:** 2013-06-14

**Authors:** Jennifer C Brennan, Michael S Denison, Dirk M Holstege, Prokopios Magiatis, Jerry L Dallas, Elisa G Gutierrez, Anatoly A Soshilov, James R Millam

**Affiliations:** 1 Department of Environmental Toxicology, University of California, One Shields Avenue, Davis, CA 95616, USA; 2 Agricultural and Natural Resources Analytical Laboratory, University of California, One Shields Avenue, Davis, CA 95616, USA; 3 Department of Pharmacognosy and Natural Products Chemistry, Faculty of Pharmacy, University of Athens, Panepistimioupolis Zografou 15 771 Athens, Greece; 4 Nuclear Magnetic Resonance (NMR) Facility Office, University of California, One Shields Avenue, Davis, CA 95616, USA; 5 Department of Chemistry, One Shields Avenue, Davis, CA 95616, USA; 6 Department of Animal Science, University of California, One Shields Avenue, Davis, CA 95616, USA

**Keywords:** *Fallopia convolvulus*, Phytoestrogens, Bioassay-directed fractionation, HPLC/MS/NMR, (−)-epiafzelechin-3-*O*-p-coumarate (rhodoeosein), Emodin, Transfection, Estrogen receptor, Relative estrogenic potency

## Abstract

**Background:**

The plant genus *Fallopia* is well-known in Chinese traditional medicine and includes many species that contain bioactive compounds, namely phytoestrogens. Consumption of phytoestrogens may be linked to decreased incidence of breast and prostate cancers therefore discovery of novel phytoestrogens and novel sources of phytoestrogens is of interest. Although phytoestrogen content has been analyzed in the rhizomes of various *Fallopia sp*., seeds of a *Fallopia sp*. have never been examined for phytoestrogen presence.

**Methods:**

Analytical chemistry techniques were used with guidance from an *in vitro* estrogen receptor bioassay (a stably transfected human ovarian carcinoma cell line) to isolate and identify estrogenic components from seeds of *Fallopia convolvulus*. A transiently transfected human breast carcinoma cell line was used to characterize the biological activity of the isolated compounds on estrogen receptors (ER) α and β.

**Results:**

Two compounds, emodin and the novel flavan-3-ol, (−)-epiafzelechin-3-*O*-p-coumarate (rhodoeosein), were identified to be responsible for estrogenic activity of *F. convolvulus* seed extract. Absolute stereochemistry of rhodoeosein was determined by 1 and 2D NMR, optical rotation and circular dichroism. Emodin was identified by HPLC/DAD, LC/MS/MS, and FT/ICR-MS. When characterizing the ER specificity in biological activity of rhodoeosein and emodin, rhodoeosein was able to exhibit a four-fold greater relative estrogenic potency (REP) in breast cells transiently-transfected with ERβ as compared to those transfected with ERα, and emodin exhibited a six-fold greater REP in ERβ-transfected breast cells. Cell type-specific differences were observed with rhodoeosein but not emodin; rhodoeosein produced superinduction of reporter gene activity in the human ovarian cell line (> 400% of maximum estradiol [E2] induction) but not in the breast cell line.

**Conclusion:**

This study is the first to characterize the novel flavan-3-ol compound, rhodoeosein, and its ability to induce estrogenic activity in human cell lines. Rhodoeosein and emodin may have potential therapeutic applications as natural products activating ERβ, and further characterization of rhodoeosein is necessary to evaluate its selectivity as a cell type-specific ER agonist.

## Background

The plant genus *Fallopia* (Polygonacae) is well known in traditional medicines, and extracts have been used to treat hepatitis, liver damage, inflammation, and postmenopausal diseases [1-4]. Compounds isolated from rhizomes of *Fallopia* sp. have demonstrated vasorelaxant, anti-oxidant, anti-bacterial, anti-inflammatory, and anti-tumor properties [4-7], which have likely led to the *Fallopia* genus being widely used in traditional Chinese medicines. Several polyphenolic compounds exhibiting estrogenic activity (phytoestrogens) have also been isolated from the roots and rhizomes of numerous *Fallopia* (recently *Polygonum*) species such as *F. multiflorum*, *F. cuspidatum*, and *F. japonica*[8-12]. Many phytoestrogens exhibit preferential activation of estrogen receptor beta (ERβ) over estrogen receptor alpha (ERα) [13], and diets high in phytoestrogen content have been correlated with lower incidence of hormone-related cancers, namely breast and prostate [14]. ERβ activation has an anti-proliferative effect in breast cells and is viewed as a protective balance against ERα activation (associated with proliferation) [15-17]. As such, there is interest in identifying plant sources rich in phytoestrogen content as well as discovering novel ERβ-selective phytoestrogens.

Recently, the rhizomes of *Fallopia convolvulus (L.) Á.Löve* (black bindweed, *Polygonum convolvulus* L. [18]) were examined for their inhibitory effects on nitric oxide production in lipopolysaccaharide-activated macrophages. Seventeen known and three novel phenolic compounds were identified in the active extract [19]. However, extracts of *F. convolvulus* have not been examined for estrogenic activity nor has the polyphenolic content of its seeds been studied. Additionally, despite the wealth of information on the polyphenolic content and/or bioactive properties in the genus *Fallopia*, no attention has been paid to the content of the seeds. *F. convolvulus* is a widely distributed species, native throughout Asia, Europe, and northern Africa and invasive in the Americas and Australia [20]. Study of ancient herb consumption in northern Europe indicates that the seeds of *F. convolvulus* were consumed by humans in early pre-Roman Iron Age and the Roman Iron Age (500 BC-400 AD) [21,22].

Several major classes of phytoestrogens exist including isoflavones, lignans, stilbenes such as resveratrol, and anthraquinones such as emodin and emodin-glycoside. Flavanols, a class rich in biologically active compounds, may undergo metabolism into ligands with estrogenic activity [23]. For identifying phytoestrogens in the *Fallopia* genus, mass spectrometry (MS), rather than diode array detection (DAD) or ultra-violet (UV) absorption, has become the method of choice due to its high specificity and ability to characterize unknowns through fragmentation, with electrospray ionization (ESI) being the predominant ionization source. The bulk of estrogenic compounds in *Fallopia* identified by MS are anthraquinones, stilbenes, and phenylpropanoids [6,9,10,24]. Fourier-transform ion cyclotron resonance (FTICR) MS has been used to determine accurate mass (and elemental composition), of estrogenic compounds [25]. ^1^H-NMR and ^13^C- NMR, have been used to elucidate the structure of many polyphenolic components from the genus *Fallopia*[19,26,27]. However, if the polyphenols contain chiral centers, NMR analysis will only yield the relative stereochemistry of the compound, and it is then necessary to use either X-ray crystallography or optical rotation combined with circular dichroism to determine the absolute stereochemistry [28]. To identify compounds with certain biologic activity in a complex matrix, toxicant identification evaluation (TIE) combines chromatography separation and bioassay analysis to achieve rapid screening, isolation, and identification of compounds of interest. TIE studies have been applied successfully to the genus *Fallopia* to isolate and identify compounds with estrogenic, antibacterial, anti-HIV, or anti-inflammatory properties ([10,29-31]). Use of the estrogen-sensitive carcinoma cell line MCF-7 guided separation of the phytoestrogens emodin and emodin 8-*Ο*-β-D-glucopyranoside from a methanolic root extract of *F. cuspidatum*[8]. These two compounds in addition to citreorosein were isolated from *F. cuspidatum* using a recombinant yeast screening assay (YES) [29]. Our objective was to determine whether the seeds of *F. convolvulus* contain compounds which display estrogenic activity (phytoestrogens), and, if so, the identity of the responsible compounds and whether they displayed ERβ-selectivity. In this study the estrogenic activity of *F. convolvulus* seed extract was evaluated using the stably transfected recombinant human ovarian carcinoma BG1Luc4E2 cell line which contains an estrogen-responsive reporter gene [32]. Through TIE, active (estrogenic) components were isolated from *F. convolvulus* seed and identified by instrumental analyses, and the transiently-transfected human breast carcinoma SKBR3 cell line was used to assess ER subtype-selectivity of the isolated estrogenic components.

## Methods

### Chemicals and standards

Restriction enzymes were purchased from New England Biolabs (Ipswich, MA), and antibodies were from Santa Cruz Biotechnology, Inc (Santa Cruz, CA). Translation grade L-[35S]-methionine (>400 Ci/mmol) was purchased from MP Biomedical (Solon, OH). Standards 17-β-estradiol (E2), emodin, and genistein were obtained from Sigma Aldrich Chemical Company (St. Louis, MO). Molecular grade dimethyl sulfoxide (DMSO) was obtained from OmniPur. Molecular grade ethanol (EtOH) and HPLC-grade solvents ethyl acetate (EtOAc) and *n*-hexane (*n*-hex) were obtained from Sigma (St. Louis, MO); HPLC-grade solvents acetonitrile (ACN), water, glacial acetic acid (HOAc), and methanol (MeOH) were obtained from Fisher Scientific (Waltham, MA). Deuterated dimethyl sulfoxide (DMSO, 99.9%) was obtained from Cambridge Isotope Laboratories (Andover, MA), and silica gel (170–400 mesh) was obtained from Fisher Scientific. Premium and charcoal-stripped fetal bovine serum (FBS) were obtained from Atlanta Biologicals (Lawrenceville, GA), Alpha Minimal Essential Medium (α-MEM) was obtained Invitrogen (San Diego, CA), Dulbecco’s Modified Eagle’s Medium (DMEM) was obtained from Sigma (St. Louis, MO), Lipofectamine 2000 transfection reagent was from Invitrogen (San Diego, CA), and Cell Culture Lysis Buffer and Passive Lysis Buffer were from Promega (Madison, WI). Protein Assay Dye Reagent Concentrate was from Bio-Rad (Hercules, CA). Seeds (catalogue numbers 11716 and 11717 for *F. convolvulus* and *F. dumetorum*, respectively) were purchased from Herbiseed Company (Twyford, United Kingdom), and species authenticity was verified by Dr. Martin Parham (Herbiseed). SKBR3 cells were purchased from ATCC (Manassas, VA). All standards were stored at 4°C +in borosilicate amber vials (Fisher Scientific) with PTFE-lined caps.

### General experimental procedures

HPLC for DAD and purification was performed using an Agilent series 1100 HPLC instrument equipped with a quaternary pump, autosampler, degasser, and Agilent Chemstation software for LC 3D systems. A Phenomenex Luna C_18_ column (150 mm × 4.6 mm I.D., 5 μm) with an Alltech guard column (Econosphere C_18_, 5 μm) was used at 23°C. LC/MS/MS analysis was performed using a triple quadrupole mass spectrometer API 2000 (PE Sciex, Concord, Ontario, Canada) operated in negative mode electrospray ionization (ESI) and a Perkin-Elmer (PE) series 200 equipped with a series 200 pump (PE), series 200 autosampler (PE), a CT0-10A column oven (Shimadzu), and DGU-14A degasser (Shimadzu) with an injection volume of 10 μL in split-injection mode. Gradient elution with a constant flow-rate of 1 mL/min was carried out with the mobile phase program outlined in Additional file 1. Ion source settings and potentials are shown in Supplementary Information. The instrument was operated with Analyst Software (v 1.3.1). FT-ICR MS analysis was performed using direct infusion into an FT-ICR MS (ThermoFisher) equipped with nano-ESI ion source, and spectra were acquired and processed with MassWorks software. Elemental composition of peaks was processed with Molecular Weight Calculator for Windows 9x/NT/2000/ME/XP (©Matthew Monroe). Emodin standard (10 μg/mL) was used as an external standard for calibrating mass accuracy of peaks in samples. NMR spectra were recorded on Bruker 600 MHz and 800 MHz Avance III spectrometers equipped with 5mm CPTXI cryogenic probes operating at 297 K. Sample volume in deuterated DMSO was 550 μL. The chemical shift (d) values are given in ppm and coupling constants (J) in Hz. Further NMR parameters are in Additional file 1. ECD spectrum was collected in MeOH using an Olis DSM 20 CD spectrophotometer and optical rotations were obtained on a Rudolph AUTOPOL IV polarimeter at wavelengths of 365, 405, 436, 546, 589, and 633 nm using a 1.0 dm cell. Specific rotations are reported in degrees per decimeter at 23°C and the concentrations are given in grams per 100 mL of solvent. Solvent used for optical rotations was MeOH (100%).

### Construction and validation of ER plasmids

The human ERβ expression plasmid (ERβ/pcDNA3) was constructed by PCR amplification of the human ERβ segment (530 aa, GenBank: AF051427.1) out of pCMV5 (kindly provided by Dr. John Katzenellenbogen, University of Illinois) and insertion of this fragment at *BstE*II and *Afl*II restriction sites into the modified pcDNA3 construct, which was previously described [33]. This construct contains a short 5′-untranslated sequence from the β-globin promoter and a 3′-untranslated region (~1.2 kb) from the mouse AhR gene that in previous studies significantly increased the *in vitro* expression of several proteins ([33] and data not shown). The human ERα expression plasmid, pcDNA3.1 + ERα, was purchased from Missouri S&T cDNA Resouce Center and was confirmed by restriction digestion. The human ERα fragment (444 aa, GenBank: AAD52984.1) from this plasmid was PCR-amplified with primers containing *BstE*II and *Afl*II sites, and the plasmid ERα/pcDNA3 was constructed by inserting this *BstE*II/*Afl*II fragment into the modified pcDNA3 vector. The generated plasmids were verified by DNA sequencing. Plasmids ERβ/pcDNA3 and ERα/pcDNA3were used as templates for *in vitro* expression (Additional file 2); ERα and ERβ were synthesized *in vitro* using the TNT Quick coupled transcription/translation rabbit reticulocyte lysate system (Promega). Briefly, ^35^S-Radiolabeled hERα and hERβ were synthesized in separate reactions *in vitro* according to manufacturer’s protocol, denatured, and subjected to SDS-polyacrylamide gel electrophoresis, and the kDa of each protein was determined by autoradiography of the dried gel.

### Cell culture and transient transfection assays

The recombinant BG1Luc4E2 cell-line containing a stably transfected estrogen-responsive luciferase reporter gene was grown and prepared for bioassay analysis as previously described [32]. Briefly, cells in 10 cm plates of approximately 20% confluence were cultured in phenol-red free DMEM supplemented with 10% charcoal-stripped FBS, for six days with daily media replacement. Cells were then plated into white, clear-bottomed 96-well tissue culture plates at a density of 750,000 cells/mL and allowed to attach for 24 h. Cells were incubated with carrier solvent EtOH (1% final solvent concentration), the indicated concentration of E2, or respective chemical or fraction treatment for 24 h at 37°C. Method blank treatments were included if applicable. After incubation cells were washed twice with PBS, followed by addition of cell lysis buffer (Promega), the plates shaken for 20 min at room temperature to allow cell lysis, and luciferase activity in each well was measured using an Orion microplate luminometer as previously described [34]. SKBR3 cells were grown and maintained in high-glucose DMEM and 10% premium FBS. SKBR3 cells were cultured in phenol red-free medium supplemented with 10% charcoal-stripped FBS for 48 h before transfection and seeded in 24-well plates at a density of 300,000 cells/mL. 24 h after plating, cells were transfected for 24 h using Lipofectamine reagent (2 μL/well) according to the manufacturer’s recommendations (0.2 μg of ER-responsive reporter plasmid pGudLuc7ere [32] and 0.05 μg receptor plasmids (ERα/pcDNA3 or ERβ/pcDNA3) or empty vector (pcDNA3.1+) per well, normalizing μg DNA/well to 0.8 μg/well with empty vector). 24 h post-transfection, SKBR3 cells were incubated for 24 h with chemical, the cells harvested, and both protein concentration and luciferase activity determined. Protein concentration was determined using the Bradford assay [35]. Briefly, 5 μL/well SKBR3 lysate was incubated for 5 min at room temperature with 1X Bradford Reagent (200 μL). Protein concentration was calibrated against a standard curve of bovine serum albumin (0.05-0.5 mg/mL) on the same plate and was measured as mg protein/mL using a PhosphorImager (Molecular Dynamics).

### Statistical analysis

Luciferase activity was expressed as relative light units (RLU) for assays using BG1Luc4E2 cells or as a ratio of luciferase RLU/mg protein for assays using SKBR3 cells. Protein amount (mg) was calculated from concentrations obtained in Bradford assay and the respective luciferase RLU was divided by mg protein to obtain luciferase RLU/mg protein. Average luciferase RLU (or luciferase RLU/mg protein) values were calculated from triplicate wells. Solvent luciferase RLU (or luciferase RLU/mg) was subtracted from luciferase RLU induced by chemical. In the case of extract and fractions, luciferase RLU induced by method blank was subtracted from luciferase RLU induced by extract or fraction. Negative values were reported as 0. Luciferase RLU and luciferase RLU/mg protein from rhodoeosein, emodin, and genistein, extracts, and fractions were calculated as a percent of maximum E2 activity (achieved with 1 nM E2 and 10 nM E2 in BG1Luc4E2 cells and SKBR3 cells, respectively, and set as 100%) as previously described [36]. The Student’s *t*-test (2-tailed, paired) was used to establish significant difference of chemical/fraction/extract activity from that of solvent or method blank (p ≤ 0.05), and dose–response curves were fitted using a sigmoidal Hill’s 4-parameter algorithm (SigmaPlot v.9.0, Systat, San Jose, CA), as described previously [37,38]. Half-maximal inductions of each treatment (EC_50_) were determined as described in [34].

### Sample extraction and isolation of (−)-epiafzelechin-3-*O*-p-coumarate

Extraction and isolation to determine the estrogenic compounds in *F. convolvulus* is described in Additional file 1. Seed was extracted using the following optimized protocol. Triple aliquots of ground *F. convolvulus* seed (25 g) were prepared. A method blank was included. Samples were sonicated in EtOAc (25 mL) for 7 min and liquid portions were collected. This process was repeated three times with an additional final solvent rinse of the samples. Liquid portions for each sample were centrifuged and re-centrifuged (550 g force) for 10 min per centrifugation. Supernatants were collected and the solvent evaporated from each sample at room temperature using nitrogen evaporation. Residues were re-suspended in EtOH and vacuum filtered using 0.2 μm Millipore filters. Solvent was evaporated and residues were re-suspended in H_2_O/ACN (1:1) 0.1% HOAc for preparative HPLC (Program 2). An isocratic program was carried out with A, water with 0.1% HOAc, and B, acetonitrile with 0.1% HOAc. The elution program was as follows: 20-30% B in 2 min (1 mL/min), 30% B for 13 min (1 mL/min), 30-100% B in 5 min (1 mL/min), with a flush of 100% B for 2 min (2 mL/min), 100-20% B in 2 min (1 mL/min), and 20% B for 1 min (1 mL/min (run-time 23 min). Valve (direct to waste) timing was 1.8 min onwards. Fractions were collected in 0.25 min intervals and evaluated for activity in BG1Luc4E2 cells, and fractions 12–14 (containing compound **5**) were combined and fractionated resulting in fractions 12–13.5 that were combined and re-fractionated to yield pure compound **5** (20.9 mg). Compound **5** ((−)-epiafzelechin-3-*O*-p-coumarate) was then evaluated for activity at different concentrations in BG1Luc4E2 cells and maximal ERα and ERβ activity in SKBR3 cells.

## Results

### Estrogenic activity of seed extracts and crude fractions

**Figure 1:**
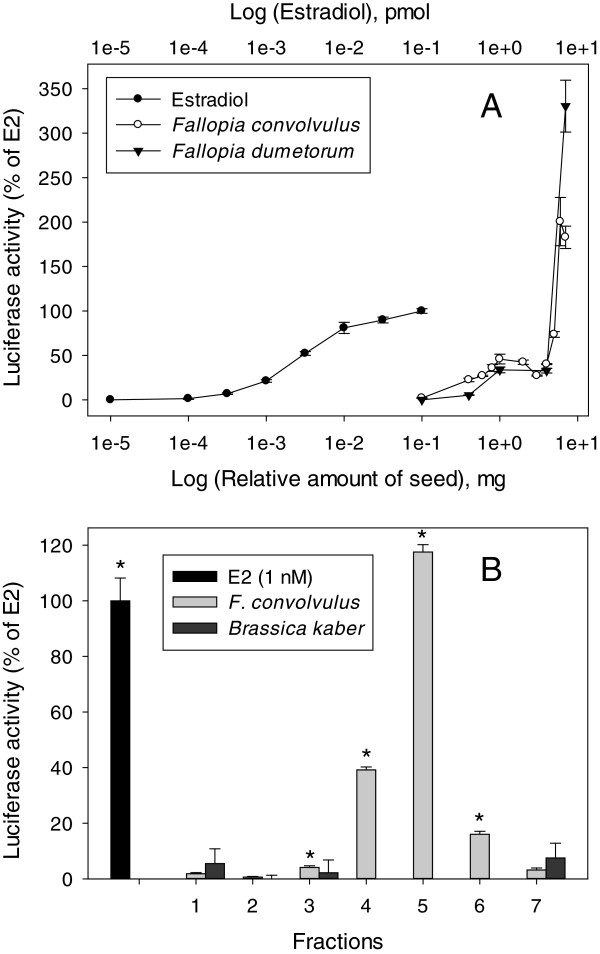
***F. convolvulus *seed induces activity in an estrogen-responsive cell line. A**. Seed extracts of *F. convolvulus* and *F. dumetorum* agonize estrogen receptor in human ovarian carcinoma BG1Luc4E2 cells. Seed (8 g) was extracted in EtOAc and BG1Luc4E2 cells were treated with indicated concentration of E2 or seed extract. Luciferase activity in cell lysates was determined after 24 h. Values represent the mean ± SD of triplicate determinations. Seed extract concentrations at and above 8 g/mL caused significant cell death and were not included in the analysis. **B** - Multiple fractions from crude chromatography of *F. convolvulus* seed (8 g/mL) exhibited greater estrogenic activity than whole seed extract in BG1Luc4E2 cells. Solvent composition of fractions 1–7 is described in Additional file 1. Activity of treatments (fractions and negative control, *Brassica kaber*) was expressed as a percent of E2 induction (1 nM). The luciferase induction profile for *F. dumetorum* crude fractions was similar to that shown here. Luciferase activity in cell lysates was measured 24 h after treatment. Values with * indicate significant difference above background using Student’s *t*-test (2-tailed, paired) and represent the mean ± SD of triplicate determinations.

*F. convolvulus* and *F. dumetorum* seeds were extracted and analyzed for estrogenic activity in human ovarian carcinoma BG1Luc4E2 cells (Figure 1A). The seed extracts of *F. convolvulus* and *F. dumetorum* superinduced ER-dependent luciferase expression at 200% and 330%, respectively, of maximal luciferase levels caused by 17β- estradiol (E2). At seed amounts equal to and greater than 8 mg on the bioassay, significant cell death was noted which suggested presence of toxins in the seed extracts (therefore values greater than 7 mg seed are not included in the analysis). The similarity in the luciferase induction pattern between *F. convolvulus* and *F. dumetorum* suggests the seeds of both species may contain very similar estrogenic/antiestrogenic components. The estrogenic properties of seeds from a *Fallopia* species have not been examined, so comparison to seeds of other *Fallopia* species is not possible. However, whole root extracts of a related species, *F. multiflorum*, were capable of inducing ER-dependent gene expression to ~75% of maximal E2 activity (from 0.1 mg root) in a yeast-based estrogen receptor assay [39] and contains an estimated 0.4 mM E2 EQ/g herb in the same receptor assay in a separate study [40]. After initial analysis on BG1Luc4E2 cells, *F. convolvulus* seed extract was then crudely fractionated using normal phase chromatography, and fractions (8 mg seed equivalents/mL) were evaluated for estrogenic activity in the BG1Luc4E2 cell line (Figure 1B). Significant luciferase induction was observed in multiple fractions, which suggested that more than one estrogenic agent may be present in the seed extract. Luciferase activity of fraction 5 alone was superinduced to 118 ± 3% seed equivalent compared to a toxic response for whole seed extract at the same concentration (Figure 1B) suggesting the fractionation removed some toxins from the whole seed extract during the crude separation. The seed estrogen(s) was/were suspected to contain both polar and nonpolar characteristics as the eluting solvent of active fractions ranged from 20% EtOAc in *n*-hex to 5% EtOH in EtOAC. Estrogenic potency values of crude fractions were calculated against an E2-dependent luciferase induction standard curve (data not shown) using the methods of Natarajan et al. [37]. Crude fractions 3, 4, and 6 of *F. convolvulus* had respective estrogenic potency values of 0.21, 2.4, and 0.76 nM E2 equivalence (eq) per g seed with 4 ± 0.6%, 39 ± 1%, and 16 ± 1%, respectively of maximal E2 activity. *Brassica kaber* seed was used as a negative control in the crude fractionation as the seed does not contain phytoestrogens.

### Isolation and identification of estrogens from *F. convolvulus* seed

**Figure 2:**
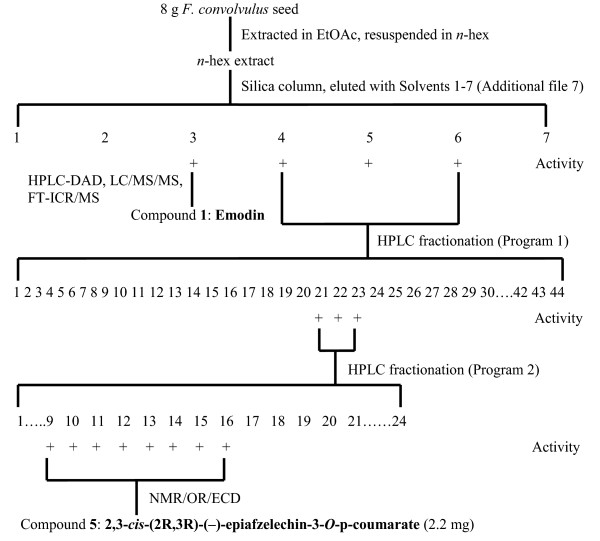
**Isolation strategy of estrogenic components from *F. convolvulus *seed.** (+) indicates activity in BG1Luc4E2 cell line. Crude chromatography is described in Additional file 1. HPLC programs 1 and 2 are described in Additional file 1 and Methods, respectively.

HPLC-DAD analysis of *F. convolvulus* seed extract showed a minimum of nine phenolic compounds (Additional files 3 and 4). The retention time and UV spectrum of the nine compounds were confirmed in *F. dumetorum* seed extract (Additional file 3). The estrogenic activity of crude fraction 3 from *F. convolvulus* seed extract was attributed to compound **1**. For isolation of the remaining estrogenic component(s), active crude fractions 4–6 of *F. convolvulus* seed were combined and subjected to HPLC fractionation (Figure 2). Using Program 1 (H_2_O/ACN gradient), 44 fractions were collected in 30 s intervals, fractions were evaporated under a stream of nitrogen, and resuspended in MeOH at a concentration of 8 g seed equivalents/mL. Fractions were screened for activity using BG1Luc4E2 cells, and active fractions (21–23) were subsequently combined and refractionated using HPLC Program 2. These second-round fractions were screened for activity, and active fractions (9–16) were verified for purity using Program 1 and combined. Compound **5**, the purified peak from these fractions, was then identified as described below. While there was an insufficient amount of compound **1** for NMR, it was identified as emodin, an anthraquinone derivative, based on retention time, UV-spectrum, mass spectrum (including MRM), and accurate mass (Additional files 1, 3, 4 and 5). In addition to the fractionation scheme showed in Figure 2, we developed an optimized isolation scheme for compound **5** (Additional file 6) in which we were able to isolate 20.9 mg compound **5** from 75 g *F. convolvulus* seed for subsequent functional analysis.

### Chemical characterization of compound 5

**Figure 3:**
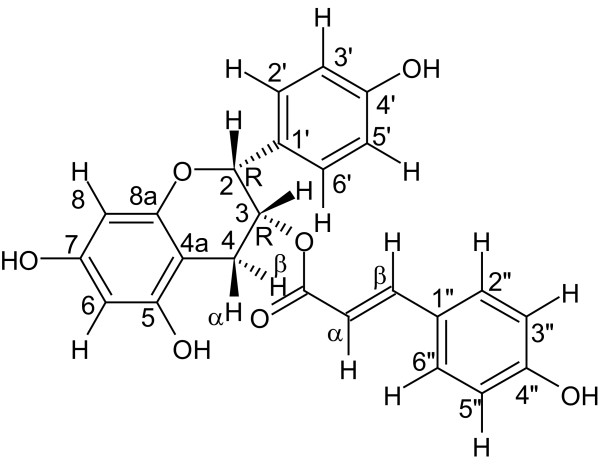
**Structure of 2,3-*cis*-(2R,3R)-(−)-epiafzelechin-3-*O*-p-coumarate (rhodoeosein), depicted in absolute stereochemistry.**

**Figure 4:**
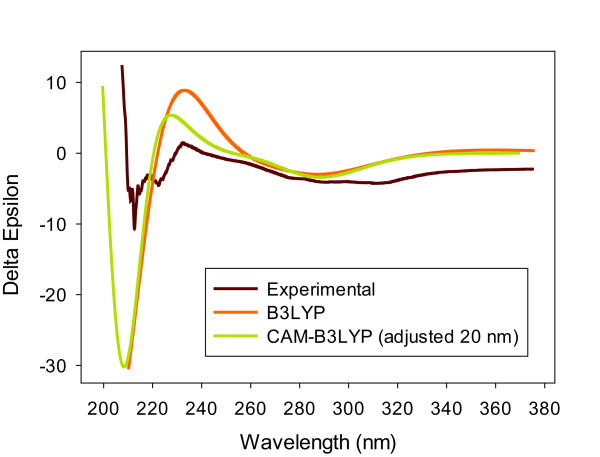
**Experimental and theoretical ECD spectrum of 2,3-*cis*-(2R,3R)-(−)-epiafzelechin-3-*O*-p-coumarate (rhodoeosein; 100 μM in MeOH).** Experimental, B3LYP, CAM-B3LYP*. *CAM-B3LYP ECD calculated prediction was adjusted +20 nm.

The purified active (estrogenic) sample, compound **5** (2.2 mg), was identified by NMR, optical rotation, and circular dichroism as 2,3-*cis*-(2R,3R)-(−)-epiafzelechin-3-*O*-p-coumarate (rhodoeosein, [Figure 3]). Table 1 shows the ^1^H and ^13^C shifts of rhodoeosein. Proton and carbon spectra and COSY, NOESY, HMBC and HSQC correlations were obtained to construct rhodoeosein in relative stereochemistry (Additional file 7). COSY correlations from NMR analysis were employed to identify vicinal relationships among the aromatic protons in each *para*-substituted phenolic ring. Coupling between H6 and H8 was also established. The *trans* Hα to Hβ relationship was based upon the magnitude of the vicinal coupling constant (15.8 Hz) which was obvious in the COSY spectrum. H3 demonstrated vicinal cross peaks to H4α and H4β. The *trans* relationship from H3 to H4β was based upon the larger vicinal coupling constant (4.9 Hz) as compared to H3 to H4α (2.4 Hz). Cross peaks in NOESY from H3 to Hα and Hβ suggest a *trans* relationship between the carbonyl and the double bond containing Hα and Hβ. A small cross peak was observed between H2 and H2’6′ thus supporting the point of attachment of one *para*-substituted phenolic ring. From the NOESY, H3 showed cross peaks to Hα and Hβ as well as H2’6′. This supported the connectivity of C3-O-C = O-α-β. Hβ revealed cross peaks to H6”2″. This supported the connectivity of Cβ to C1”. H8 was differentiated from H6 by cross peaks to H2′ and H2. Thus H6 was more distant from either phenolic ring. A weak cross peak from H4α was observed to Hβ and Hα (not shown). From HMBC, H3 showed a fundamental correlation to C = O further supporting the connectivity of C3-O-C = O. Hβ yielded cross peaks to C2”6″ indicating the attachment of Cβ to C1”. Cross peaks from H2 to C2’6′ indicated the connectivity of C2 to C1’. Other important correlations were revealed with cross peaks from H4α and H4β to C4a and C8a. H8 showed correlations to C7 and C8a. Also, H6 exhibited correlation to C4a, C7 and C5. The combination of this information led to the unique assignments of carbons in the bicyclic ring (C2-C8a) and also supported the connectivity of the remaining moieties. Resolution of the HMBC experiment did not permit differentiation between C5 and C7. The resonances were separated by only 0.1 ppm. The assignments of these two resonances could be reversed without impacting any other assignments or the proposed structure. Regarding the absolute stereochemistry, rhodoeosein contains two stereogenic centers (C2, C3, Figure 3). As the majority of *cis* flavan-3-ols from natural sources exist in the 2R,3R conformation [41,42], it was not surprising that optical rotation combined with electronic circular dichroism (ECD) indicated the absolute stereochemistry of rhodoeosein as 2R,3R (Figure 4). Optical rotation values were negative at all tested wavelengths at two different concentrations of rhodoeosein in MeOH (Additional file 7) which is in agreement with the parent compound, (2R,3R)-(−)-epiafzelechin [43]. ECD spectra were calculated for the 2R,3R conformation of rhodoeosein using B3LYP and CAM-B3LYP predictions and compared to the experimental spectrum (Figure 4). The overlay of experimental ECD spectrum on predicted spectra indicated good agreement for a 2R,3R confirmation of rhodoeosein. The predicted ECD calculations (the CAM-B3LYP prediction was adjusted +20 nm) for the 2R,3R confirmation of rhodoeosein showed a positive L_a_ transition at ca.230 nm (corresponding to 3R), matching that of the experimental ECD (Figure 4) and of the Boltzmann-weighted parent compound [43-45]. The L_b_ transition for the experimental spectrum of rhodoeosein occurred at ca.300-310 nm whereas the L_b_ transition for the predicted ECD spectra of the 2R,3R confirmation of rhodoeosein occurred at ca.290 nm; however both predicted and experimental spectra showed a negative L_b_ transition which corresponds to an R conformation at C2 [41] (it should be noted that both the experimental and calculated L_b_ transitions for rhodoeosein were outside the traditional range of the L_b_ transition for flavan-3-ols which is 260–280 nm [46]). The parent compound, (2R,3R)-(−)-epiafzelechin, also displays a negative L_b_ transition [43-45]. From the mass spectrum of rhodoeosein, a parent peak and a dimer of the parent peak were noted at 419 m/z ([M-1]) and 839 m/z ([2M-1]), respectively (Additional file 7). Fragment peak 145 m/z was interpreted as the 3-O-p-coumarate portion of rhodoeosein (with the remainder of the molecule as fragment 273 m/z).

**Table 1:** ^**1**^**H and **^**13**^**C NMR spectroscopic data of compound 5 (600 MHz, δ in ppm)**

**Atom #**	^**1**^**H**		^**13**^**C**
2	5.07	(s)	76.3
3	5.34	(m)	67.8
4α	2.65	(dd, J = 17.6, 2.4 Hz)	25.6
4β	2.92	(dd, J = 17.6, 4.9 Hz)	25.6
4a			97.2
5			156.6*
6	5.94	(d, J = 2.4 Hz)	95.6
7			156.5*
8	5.79	(d, J = 2.4 Hz)	94.3
8a			155.5
1'			128.6
2',6'	7.25	(d, J = 8.6 Hz)	127.8
3',5'	6.7	(d, J = 8.6 Hz)	114.8
4'			156.9
C = O			166.1
α	6.25	(d, J = 15.8 Hz)	113.6
β	7.38	(d, J = 15.8 Hz)	145.3
1"			124.6
2",6"	7.5	(d, J = 8.6 Hz)	130.5
3",5"	6.72	(d, J = 8.6 Hz)	115.8
4"			160.3

Compound was measured in DMSO. Chemical shifts are reported relative to TMS (residual dimethylsulphoxide-D6 = 2.50 ppm for ^1^H and 39.51 ppm for ^13^C). *Chemical shift assignment for C5 and C7 may be reversed. HMBC data did not permit resolution sufficient to unambiguously assign the two resonances. This finding has no impact on the proposed structure. A single broad –OH resonance was observed at 9.9 ppm.

#### 2,3-cis-(2R,3R)-(−)-epiafzelechin-3-O-p-coumarate (rhodoeosein)

Translucent pink oily film from 70/30 H_2_O/ACN; pink in MeOH and EtOH; [α]_23 D_ -151 (*c* 0.22, MeOH); For ^1^H and ^13^C NMR spectroscopic data, see Table 1; for CD data (MeOH), see Figure 4; ESIMS m/z: 840.5 [2M], 419.4 [M-1], 273, 254.9, 229.1, 164.9, 145: 419.11360 [M-H]^-^ (C_24_H_19_O_7_, experimental 419.11322, calc 419.11307). For peak abundances, UV spectrum, HMBC, HSQC, NOESY, and COSY spectra, see Additional file 4: Table S1 and Additional file 7.

### Biological characterization of rhodoeosein and emodin

**Figure 5:**
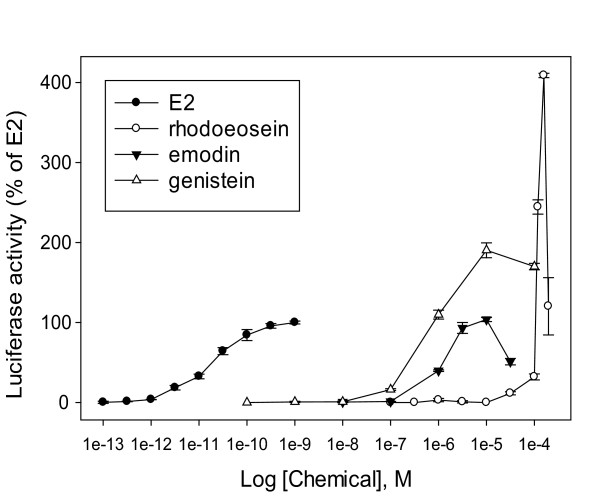
**Potency curves of phytoestrogens rhodoeosein and emodin in human ovarian carcinoma BG1Luc4E2 cells.** ●= E2; ○ = rhodoeosein; ▼ = emodin; Δ = genistein. Rhodoeosein was able to superinduce luciferase activity in BG1Luc4E2 cells. Luciferase activity was expressed as a percent of the maximum induction by E2 (1 nM) and was determined in cell lysates 24 h after treatment. Incubation of cells with 0.25, 0.316, and 1 mM rhodoeosein caused cell death and these values were not included in above analysis. Values represent the mean ± SD of triplicate determinations.

To characterize the estrogen activity of the novel phytoestrogen, rhodoeosein, we analyzed its activity over a range of concentrations in two human cell lines: a recombinant human ovarian carcinoma (BG1Luc4E2) cell line [32] and a human breast carcinoma (SKBR3) cell line. In addition to E2, we also compared the activity of rhodoeosein with that of emodin and of a well-known phytoestrogen control, genistein. Both rhodoeosein and emodin induced luciferase induction in the stably-transfected BG1Luc4E2 cells. Rhodoeosein had a narrow range of luciferase-inducing concentrations in BG1Luc4E2 cells (Figure 5); incubation with rhodoeosein below 31.6 μM resulted in no luciferase induction whereas incubation with 119 μM and 158 μM rhodoeosein resulted in superinduction of ER-dependent luciferase in BG1Luc4E2 cells (> 400% of maximum E2 activity). Incubation with rhodoeosein at concentrations at or above 250 μM killed BG1Luc4E2 cells (no toxicity was observed at 197 μM rhodoeosein). Based on maximum luciferase induction at 158 μM rhodoeosein, EC_50_ of rhodoeosein was 120 μM ± 0.360 μM rhodoeosein, approximately 7 orders of magnitude weaker in potency than the E2 standard (Figure 5, Table 2). As a comparison, emodin displayed half-maximal activity in BG1Luc4E2 cells at 1.3 μM emodin, approximately 5 orders of magnitude weaker in potency than the E2 standard (EC_50_ 18 pM ± 9.4 nM) and was able to induce luciferase to 104 ± 3% of maximum E2 activity (Figure 5, Table 2). Emodin’s estrogenic properties have been previously demonstrated in a recombinant yeast screening assay (YES) containing ERα; emodin was only two orders of magnitude weaker in estrogen response than the E2 standard [12]. When assessing genistein’s activity in BG1Luc4E2 cells, we found genistein was able to superinduce luciferase (190 ± 9% of maximum E2 activity) and was, not surprisingly, more potent than either rhodoeosein or emodin with half-maximal induction at 0.73 μM ± 0.15 μM (approximately 4 orders of magnitude weaker than the E2 standard).

**Table 2:** Potency comparisons of phytoestrogens

	**EC**_**50 **_**(M), REP**			
**Cell-line**	**E2**	**Rhodoeosein**	**Emodin**	**Genistein**
BG1Luc4E2	1.8 × 10^-11^, 1	1.2 × 10^-4^, 3.5 × 10^-7^	1.3 × 10^-6^, 1.5 × 10^-5^	7.3 × 10^-7^, 2.5 × 10^-5^
SKBR3 (ERα)	2.0 × 10^-11^, 1	5.8 × 10^-6^, 4.0 × 10^-6^	1.3 × 10^-6^, 1.5 × 10^-5^	9.0 × 10^-7^, 2.2 × 10^-5^
SKBR3 (ERβ)	2.7 × 10^-10^, 1	1.8 × 10^-5^, 1.8 × 10^-5^	3.0 × 10^-6^, 8.9 × 10^-5^	8.3 × 10^-8^, 3.3 × 10^-3^

Comparison of concentration for half-maximal induction (EC_50_) and relative estrogenic potency (REP) for E2, rhodoeosein, and emodin in human carcinoma cell-lines BG1Luc4E2 (ovarian) and SKBR3 (breast) transfected with either ERα or ERβ. Data for genistein is included for comparative purposes. REP = EC_50_ E2/EC_50_ chemical.

**Figure 6:**
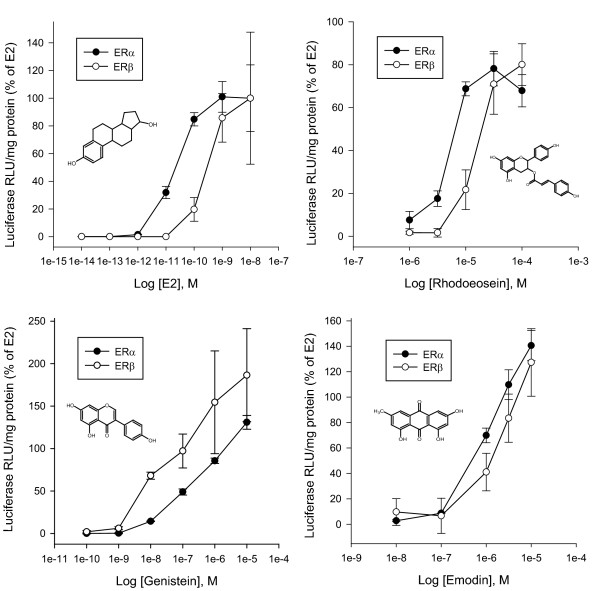
**Phytoestrogens rhodoeosein and emodin isolated from *F. convolvulus *seed induce luciferase expression in human breast carcinoma SKBR3 cells transiently transfected with ERα or ERβ.** ●= ERα-transfected SBKR3 cells; ○ = ERβ-transfected SKBR3 cells. Luciferase activity was normalized with protein concentration, was expressed as a percent of the maximum induction by E2 (10 nM), and was determined in cell lysates 24 h after treatment. Values represent the mean ± SD of triplicate determinations. Incubation of SKBR3 cells with 0.316 mM rhodoeosein caused cell death and was not included in the above analysis.

Although the BG1Luc4E2 cell line endogenously expresses ERα, it does not appear to express ERβ protein [32]. Given the critical role that ERβ plays in normal physiological/endocrinological processes and tissue health and in modulating the functional activity and levels of ERα [47-50], interest in ligands that selectively interact with ERβ has grown. To evaluate activity of rhodoeosein and emodin on both ER subtypes, rhodoeosin was incubated with SKBR3 cells transiently co-transfected with an estrogen-response firefly luciferase reporter plasmid [32] and either an ERβ (ERβ/pcDNA3) or ERα (ERα/pcDNA3) expression plasmid. Firefly luciferase activity in each well was normalized to protein amount and the resulting ratio expressed as a percent of maximum induction by E2 (10 nM). In contrast to the results BG1Luc4E2 cells, rhodoeosein did not superinduce ER-dependent gene expression in SKBR3 cells transfected with either ER subtype; maximum luciferase induction by rhodoeosein was 78 ± 8% and 80 ± 10% of E2 activity in SKBR3 cells transfected with either ERα or ERβ, respectively (Figure 6). Concentrations of rhodoeosein greater than 100 μM rhodoeosein resulted in significantly decreased protein concentration in SKBR3 cells and are not included in the analysis. Half-maximal induction (EC_50_) by rhodoeosein in ERα- and ERβ-transfected SKBR3 cells was 5.8 μM ± 1.5 μM and 18 μM ± 0.4 μM, respectively (significantly less potent than E2 which had EC_50_ values of 20 pM ± 1.5 nM and 270 pM ± 4.0 nM in ERα- and ERβ-transfected SKBR3 cells, respectively). The phytoestrogen control, genistein, superinduced at 10 μM genistein in ERα-transfected cells, but did not significantly superinduce in ERβ-transfected cells. Genistein was the only phytoestrogen in this study to exhibit a lower EC_50_ value in ERβ-transfected SKBR3 cells than in ERα-transfected SKBR3 cells (EC_50_ values 83 ± 120 nM and 0.90 ± 0.88 μM, respectively). Concentrations of genstein greater than 10 μM resulted in significantly decreased RLU in SKBR3 cells and are not included in the analysis. Emodin was also evaluated in ER-transfected SKBR3 cells; maximum induction by emodin was significantly greater than that of E2 in ERα-transfected but not ERβ-transfected SKBR3 cells with 141 ± 12% and 127 ± 26% of maximum E2 activity, respectively (Figure 6). Concentrations of emodin greater than 10 μM were toxic to SKBR3 cells and therefore are not included in the analysis. EC_50_ values for emodin in ERα-transfected and ERβ-transfected SKBR3 cells were 1.3 μM ± 0.24 μM and 3.0 μM ± 1.0 μM, respectively, (as compared to emodin’s EC_50_ value of 1.3 μM in BG1Luc4E2 cells). While emodin was a more potent phytoestrogen than rhodoeosein for both ERα and ERβ, it is present at much lower concentrations in the seed (Additional file 4), and as such, rhodoeosein likely is responsible for a greater amount of the estrogenic activity of whole seed.

## Discussion

Consumption of a diet rich in phytoestrogens has been strongly correlated with beneficial effects on human health [51,52]. Flavan-3-ols (the subclass of compounds containing rhodoeosein) have been reported to have a wide array of positive health effects such as antioxidant, anti-viral, anticarcinogenic, antimicrobial, and cardiopreventation (reviewed in [53]). Aside from the current study, there is no report of phytoestrogenic activity associated with flavan-3-ols. However, flavonoids, the broad class of plant compounds under which flavan-3-ol is found, contains many phytoestrogens (genistein, biochanin A, daidzein). Thorough structure-function analyses of various flavonoids on estrogen-responsive cell lines have correlated hydroxyls at the 7 and 4′ positions in flavonoids to those at the 17 and 3 positions in E2 and concluded that the flavonoid hydroxyls are essential for estrogenic activity [54,55]. The estrogenic flavonoids in these two studies contained between 2 to 4 hydroxyl substituents. Accordingly, it is not surprising that rhodoeosein is estrogenic since it contains hydroxyls at the 7 and 4′ positions (Figure 3) and displays the classic A-ring structure which is shared by all known estrogenic compounds [56]. A further phenomenon that may allow for the estrogenic activity of rhodoeosein is increased acidity of a hydroxyl through in-plane hydrogen bonding as suggested for other compounds by Fang et al. [56]. Rhodoeosein possesses a benzylic hydrogen, H_β_, that is in close proximity to an ester on C = Ο (in conjugation with a hydroxyl in the 4″ position). This ester may be able to form an intramolecular H-bond with the in-plane C4 H_β_ (Figure 3); the increased electron-withdrawing capability of the C = Ο carbonyl from an H-bond interaction with the C4 H_β_ would increase the acidity of the 4″ hydroxyl (favorable for ER activity). In Zand et al. [55], a double bond between carbons 2 and 3 was important for estrogenic activity of a flavonoid as the double bond at this position increased rigidity of the molecule and, therefore, ER-affinity [56]; interestingly, rhodoeosein lacks a double bond at this position in the molecule but contains, in addition to the A- and B-rings, a large conjugated system exhibiting rigidity (−O-p-coumarate) which may lend favorable ER-binding attributes to the molecule. It may be that the structurally-favorable aspects of rhodoeosein (hydroxyls at the 7 and 4′ positions, having 4 hydroxyl substituents, rigidity) outweigh the absence of a double bond between C2 and C3. Additionally, molecules exhibiting a certain measure of hydrophobicity (where the molecule is hydrophobic while containing a polar group on each end) bind to ER with greater affinity [56]. Rhodoeosein contains not just one set of opposing polar groups but two (7-OH and 4′-OH, 7-OH and 4″-OH) which sandwich large hydrophobic regions (Figure 3).

Plants have a complex pathway for flavan-3-ol formation. Flavan-3-ols can be found in the seeds and fruits but not leaves of certain plants, and a pathway for flavonoid conversion in the plant from flavanone to flavan-3-ol has been proposed [45]. The gene responsible for the formation of flavan-3-ol through this cascade is highly expressed in the seeds but not in the flowers or leaf tissues [45]. Recently, *F. convolvulus* roots were examined for polyphenolic content [19]. Except for emodin, none of the 20 polyphenolic compounds (3 new, 17 known) found in the roots seem to exist in the seeds of *F. convolvulus* (based on mass spectrometry supplementary data). This curious phenomenon of the polyphenolic content of the seeds being unrepresentative of the content in the roots (aside from emodin) may be explained through the multi-step flavonoid pathway illustrated in Xie et al. [45]. It is thought that flavan-3-ols (known as tannins in plants) play a mainly defensive role in the plant against herbivorous animals, insects, fungi, and other harmful agents [53]. However, as demonstrated here and in other studies, flavan-3-ols exhibit complex biological properties in human systems.

Although emodin’s behavior in ERα-transfected SKBR3 cells and BG1Luc4E2 cells was consistent (Table 2), rhodoeosein showed marked cell-specific differences in the potency and magnitude of luciferase induction between BG1Luc4E2 cells and ERα-transfected SKBR3 cells (Table 2). Luciferase activity was induced by rhodoeosein in BG1Luc4E2 cells to a level greater than that maximally induced by E2 and this superinduction response suggests that rhodoeosein is also affecting other signaling pathways that impact on ER-dependent activation of luciferase expression from the reporter plasmid. Numerous studies have not only demonstrated that the functional activities of ER and other steroid hormone receptors can be altered by posttranslational modifications (i.e. phosphorylation, acetylation and others), but phosphorylation can also activate the transcriptional activity of these receptors in the absence of ligand binding [57-60]. Binding of ligand to a cytosol-membrane ERs or to GPR30 (a G-coupled protein receptor) can activate protein-kinase cascades which phosphorylate and activate nuclear ERs as well as other signaling pathways and transcription factors [57]. For example, tectoridin, a flavonoid phytoestrogen, was found to exert its estrogenic effects not through the ER but through an extracellular signal-regulated kinase pathway [61]. Additionally, direct or indirect stimulation of growth factor receptors can lead to activation of protein-kinase cascades and hormone receptor-dependent and independent gene expression responses. The enhancement of reporter gene expression observed with rhodoeosein treatment in BG1Luc4E2 cells but not SKBR3 cells could result from a rhodoeosein-dependent stimulation of an additional signaling pathway(s) that enhances the transcription of the luciferase reporter gene promoter. This pathway(s) may not present or affected in the SKBR3 cells. Although superinduction has been previously observed in the BG1Luc4E2 cell line [38], the molecular mechanism responsible for superinduction of ER-dependent gene expression in BG1Luc4E2 cells remains to be elucidated.

Regarding half-maximal concentration differences between the two ER subtypes, we did not find that rhodoeosein or emodin had lower EC_50_ values in ERβ-transfected SKBR3 cells than in ERα-transfected SKBR3 cells which does not agree with previous literature on phytoestrogens [13,62]. However, the ERβ-transfected SKBR3 cells appear to be an order of magnitude less responsive to ER-ligands than the ERα-transfected SKBR3 cells as shown by the EC_50_ values of E2, rhodoeosein, and emodin in both transfections (Table 2). Therefore, we choose to assess potency by comparing the EC_50_ values of emodin and rhodoeosein directly to those of the E2 standards in the ERα-transfected cells and the ERβ-transfected cells (Table 2). This method of using relative estrogenic potency (REP) has been previously established in [63] where REP was defined as the ratio between E2 EC_50_ and EC_50_ of the chemical. We have also defined REP = EC_50_ of E2/EC_50_ of phytoestrogen, and when compared to the half-maximal concentration of the E2 standard, both emodin and rhodoeosein were more potent in ERβ-transfected SKBR3 cells than in ERα-transfected SKBR3 cells (Table 2). REP of rhodoeosein in ERα-transfected SKBR3 cells was 3.4 × 10^-6^ but in ERβ-transfected SKBR3 cells was 1.5 × 10^-5^, a 4-fold increase in REP. The difference between emodin’s REP values in ERα-transfected SKBR3 cells and ERβ-transfected SKBR3 cells was similar to that of rhodoeosein; REP of emodin in ERβ-transfected SKBR3 cells was 8.9 × 10^-5^ whereas in ERα-transfected SKBR3 cells, the REP was 1.5 × 10^-5^, a 6-fold decrease. This is in agreement with [62] where A YES assay transfected separately with ERα or ERβ was used to examine emodin’s estrogenic activity; emodin had greater REP in ERβ-transfected yeast cells than in ERα-transfected yeast cells by approximately one order of magnitude. Our findings are also in agreement with a study examining binding affinity of several phytoestrogens to either ERα or ERβ; compared to the E2 standard (set as 100%), relative binding affinity of genistein, biochanin A, coumestrol, and diadzein were greater to ERβ than to ERα [64]. In our assay, the control phytoestrogen genistein showed a 146-fold increase in REP for ERβ compared to ERα (REP values 3.3 × 10^-2^ and 2.2 × 10^-5^, respectively), in agreement with [63]. ERα and ERβ have unique and overlapping tissue distribution in the human body, and the roles of the ER subtypes in the human body are now becoming more clear (reviewed in [65] and [52]). Although ERβ activation may be associated with certain deleterious effects (i.e. potential involvement in metabolic disorders leading to diabetes [65]), beneficial roles of ERβ in the human body include development and maintenance of the brain, ovulation, prostate health, and anti-proliferative roles in certain breast cancers. The effectiveness of phytoestrogens, which exist in nature not as single compounds but as complex mixtures in food matrices, as therapeutic agents for existing diseases is still unclear, but epidemiological studies indicate diets containing a significant amount of phytoestrogens seem to correlate with ERβ-mediated benefits (such as decreased incidence of breast and prostate cancers). The higher REP values displayed by rhodoeosein and emodin in ERβ-transfected cells may indicate use of foods containing these compounds as part of a comprehensive plan for maintaining tissue health through ERβ-mediated activity.

## Conclusion

Seeds of *F. convolvulus* were identified as a novel source of phytoestrogens from which we have isolated and chemically characterized a novel phytoestrogen rhodoeosein. Estrogenic activity of rhodoeosein was evaluated in two human cell-lines in which we were able to demonstrate cell-type specific effects of rhodoeosein. To our knowledge, rhodoeosein is the first published flavan-3-ol to demonstrate estrogenic properties *in vitro*. In addition to rhodoeosein, we also found that *F. convolvulus* seed contains emodin (a known and potent estrogen), and these compounds were also identified in the closely-related species *F. dumetorum*. By comparing relative estrogenic potencies (REP) of emodin and rhodoeosein in SKBR3 cells transfected with either ERα or ERβ we found that both phytoestrogens are more potent in ERβ-transfected cells than in ERα-transfected cells and that emodin is more potent than rhodoeosein on both ER subtypes. Similar differences in potency were observed in the BG1Luc4E2 cell line. The estrogenic compounds in this study may regulate reproduction to some degree in wildlife consuming these seeds [38]. Effects *in vivo* have not yet been assessed, but the complex polyphenolic content of *F. convolvulus* seed may indicate therapeutic potential.

## Abbreviations

ER: Estrogen receptor; MS: Mass spectrometry; DAD: Diode array detection; UV: Ultra-violet; ESI: Electrospray ionization; FTICR: Fourier transform ion cyclotron resonance; NMR: Nuclear magnetic resonance; CD: Circular dichroism; ECD: Electronic circular dichroism; TIE: Toxicant identification evaluation; EC50: Effective concentration 50; MRM: Multiple reaction monitoring; eq: Equivalence; HMBC: Heteronuclear Multiple Bond Correlation; COSY: Correlation spectroscopy; NOESY: Nuclear Overhauser effect spectroscopy; HSQC: Heteronuclear Single Quantum Coherence; REP: Relative estrogenic potency.

## Competing interests

The authors declare that they have no competing interests.

## Authors’ contributions

JM and JB designed the experiment with significant input from MD and DH. JB did fractionation (isolation), HPLC-DAD analyses, bioassay analyses, and drafted the paper. MD, DH, and PM provided valuable expertise and advice on different steps of the project. DH did HPLC/MS/MS analyses and advised on FT-ICR/MS analyses. JD ran NMR analysis of rhodoeosein. PM elucidated rhodoeosein’s structure and relative stereochemistry. JD confirmed rhodoeosein’s structure elucidation, constructed Table 1, Figure 3, and all supplemental NMR figures regarding rhodoeosein. EG did ECD analysis for rhodoeosein. AS designed ER/pcDNA vectors and did *in vitro* expression. All authors provided feedback to JB during the drafting process. All authors read and approved the final manuscript.

## Pre-publication history

The pre-publication history for this paper can be accessed here:

http://www.biomedcentral.com/1472-6882/13/133/prepub

## Supplementary Material

Additional file 1 **Supplemental text.** Further details of the isolation procedure, HPLC program 1, and NMR parameters. Click here for file

Additional file 2 **Validation of ER plasmids and the SKBR3 cell line.** In vitro expression of the ER/pcDNA3 plasmids and a control transfection experiment using SKBR3 cells. Click here for file

Additional file 3 **HPLC chromatograms of *F. convolvulus *seed and crude fractions.** HPLC chromatograms of active (estrogenic) crude fractions and whole seed extracts. Click here for file

Additional file 4: Table S1 Retention times, relative abundance, and elemental composition of pure standards and polyphenolic compounds of interest from *F. convolvulus* seed (shown as chomatographic peaks 1-9 which correspond to compounds 1-9). **Table S2.** Validation of HPLC-DAD method. **Table S3.** Declustering potential (DP), Ionization potential (FP), Entrance potential (EP), Collision Energy (CE), Collision cell entrance potential (CEP), Collision cell exit potential (CXP) and dwell time of selected transitions for the emodin standard. Click here for file

Additional file 5 **Comparison of compound 1 (in *F. convolvulus *seed) to emodin standard.** UV spectrum, mass spectrum, and MRM transitions of compound1 (emodin). Click here for file

Additional file 6 **Optimized fractionation scheme for isolation of 2,3-cis-(2R,3R)-(−)-epiafzelechin-3-O-p-coumarate from *F. convolvulus *seed.** Click here for file

Additional file 7 **Chemical characterization data for compound 5 (2,3-cis-(2R,3R)-(−)-epiafzelechin-3-O-p-coumarate [rhodoeosein]).** UV-spectrum, mass spectra, optical rotation, and NMR spectra and correlations of rhodoeosein (isolated from *F. convolvulus* seed). Click here for file
