# Supplementary material for: 2,3-cis-2R,3R-(−)-epiafzelechin-3-O-p-coumarate, a novel flavan-3-ol isolated from Fallopia convolvulus seed, is an estrogen receptor agonist in human cell lines
Source: BMC Complement Altern Med. 2013 Jun 14;13:133. doi: 10.1186/1472-6882-13-133 (PMC3695784; doi:10.1186/1472-6882-13-133)
Supplement: Additional file 1 — Supplemental text. Further details of the isolation procedure, HPLC program 1, and NMR parameters. [file 1472-6882-13-133-S1.pdf]

Isolation of estrogenic components from F. convolvulus seed

F. convolvulus seed (8 g) was ground to silty consistency. Ground *Brassica kaber* seed (8 g) was included as negative control and a method blank was included. Samples were sonicated in EtOAc (10 mL) for 10 min and liquid portions were collected. This process was repeated three times with an additional final solvent rinse of the samples. Liquid portions for each sample were centrifuged and re-centrifuged (550 g force) for 10 min per centrifugation. Supernatants were collected and the solvent evaporated from each sample at room temperature using nitrogen evaporation. Residues were re-suspended in *n*-hex for immediate application to crude chromatography columns; extracts were loaded on silica gel columns preconditioned with *n*-hex with a silica volume of 63 cm^3 (column volume 350 cm^3) at room temperature and atmospheric pressure. Each sample (extract) was eluted as seven 50 mL fractions and collected (F1: *n*-hex, F2: *n*-hex-EtOAc [95:5], F3: *n*-hex-EtOAc [80:20], F4: *n*-hex-EtOAc [50:50], F5: EtOAc, F6: EtOAc-EtOH [95:5], F7: EtOH). Crude fractions were evaporated to dryness and re-suspended in the respective solvent (1 mL), and the ability of a crude fraction (1 μL fraction per well) to induce luciferase activity in the BG1Luc4E2 cell line was determined as described in Materials and Methods, with fractions 3-6 exhibiting activity. The estrogenic activity of fraction 3 was attributed to compound **1** (based on chromatogram in Additional File 3). For isolation of the remaining estrogenic component(s), active fractions 4-6 from the crude extract of *F. convolvulus* seed were combined, resuspended in 80/20 H_2O:ACN (0.1% HOAc), and subjected to HPLC fractionation. Using the HPLC program described below (Program 1), fractions were collected in 30 s intervals in 7 mL glass vials. Fractions were solvent-evaporated using nitrogen evaporation and resuspended in methanol (8 g seed equivalence/mL). Fractions of each sample were screened for activity using BG1Luc4E2 cells and active fractions (21-23) were combined and

refractionated using the HPLC program described in Materials and Methods (HPLC Program 2). Second-round fractions were screened for estrogenic activity; active fractions (12-13.5) were verified for purity and combined (presence of purified compound **5** was confirmed using HPLC-DAD program described in Materials and Methods). Purified compound **5** (2.2 mg) was identified by FT-ICR-MS, NMR, optical rotation, and circular dichroism as 2,3-*cis*-(2R,3R)-(–)-epiafzelechin-3-*O*-p-coumarate (rhodoeosin). There was not sufficient amount of compound **1** for NMR; however, compound **1** was identified as emodin based on retention time, mass spectrum, and accurate mass ($C_{15}H_9O_5$, 269.04648 experimental, 269.04500 calc).

FT-ICR-MS calibration

Experimental accurate mass of Compounds 1 and 5 were adjusted based on the error of the emodin standard’s accurate mass (269.04638 m/z experimental, 269.04500 m/z expected, 5.14 ppm error). Elemental compositions were determined from adjusted experimental accurate masses using Molecular Weight Calculator.

HPLC program 1

A Phenomenex Luna C_{18} column (150 mm x 4.6 mm I.D., 5 μm) with an Alltech guard column (Econosphere C_{18}, 5 μm) was used at room temperature with an injection volume of 5 μL (75 μL for fraction collection). Gradient elution was carried out with A, water with 0.1% HOAc, and B, acetonitrile with 0.1% HOAc. The elution program was as follows: 20-100% B in 35 min (1 mL/min), 100% B for 5 min (2 mL/min), 100-20% B for 5 min (1 mL/min), 20% B for 5 min (1 mL/min) with a run-time of 25 min. The syringe was flushed with MeOH pre-injection and post-injection for each sample. Valve (direct to waste) timing was 1.8 min onwards. Signals

were monitored (280 nm and 320 nm) using an Agilent 1100 diode array detector, and spectra was collected (200 nm to 420 nm) with a peak threshold of 1.0 mAU. All samples subject to HPLC analysis were dissolved in MeOH. Instrumental detection limit (LOD) and instrumental quantitation limit (LOQ) for emodin was calculated for the HPLC-DAD using a signal to noise (S/N) ratio of 3/1 for the LOD and 10/1 for the LOQ. Intraday and interday variation (% RSD) was analyzed on HPLC-DAD by repeat injections of emodin standard (10 μg/mL, n = 4). To determine extraction efficiency, triplicate spiked samples of *F. dumetorum* seed were prepared by adding 1 mL of 10 μg/mL emodin standard in MeOH to each spike sample (2.5 g). Spiked samples and a check sample were extracted in EtOAc as described above.

LC/MS/MS settings

Ion source settings were as follows: nebulizer gas (N_2) 8 (psi) and 350°C, curtain gas (N_2) 45 (psi), ion source gas 1 (N_2) 60, ion source gas 2 (N_2) 15, collision gas (N_2) 6 and ionspray voltage 4500 V. DP, EP and CE were optimized for standards in direct infusion experiments. Emodin standard (10 μg/mL) dissolved in MeOH-H_2O, 0.1% HOAc (75:25) was infused at a constant flow rate of 10 μL/min into the mass spectrometer using a Waters 510 pump. Full scan data were acquired in negative ion mode by scanning 100 *m/z* to 600 *m/z* and 100 *m/z* to 1000 *m/z* with a cycle time of 2 sec/scan (DP 70 V, FP 400 V, EP 10.50 V, and CEP -11.02-33.52 V). Multiple reaction monitoring (MRM) was used to monitor several transitions for each standard on $[M - H]^-$ (dwell times in Table 3S). Precursor-ion and product-ion scan data were acquired in negative ion mode.

NMR parameters for (–)-epiafzelechin-3-O-p-coumarate

cosygpqf [acq 2K x 128, ZF to 2K x 2K, NS=4, SW=8ppm, repetition rate 6.2 sec, total time 50min]

noesygpphpp [acq 2K x 256, ZF to 2K x 2K, NS=16, repetition rate 7 sec, mix time 800 ms, SW=8ppm, sinesquarebell apodization(0 deg),total time 8 hrs]

hsqcgpph [acq 2K x 256, ZF to 2K x 1K, NS=16, repetition rate 6.2 sec, SW= 8 x 162 ppm, sinesquarebell apodization(90 deg), total time 5 hrs]

hmbcgplpndqf [acq 2K x 128, ZF to 2K x 2K, NS=32, repetition rate 6.2 sec, SW= 8 x 162 ppm, sinesquarebell (60 deg), total time 7 hrs]
