# Supplementary material for: 2,3-cis-2R,3R-(−)-epiafzelechin-3-O-p-coumarate, a novel flavan-3-ol isolated from Fallopia convolvulus seed, is an estrogen receptor agonist in human cell lines
Source: BMC Complement Altern Med. 2013 Jun 14;13:133. doi: 10.1186/1472-6882-13-133 (PMC3695784; doi:10.1186/1472-6882-13-133)
Supplement: Additional file 2 — Validation of ER plasmids and the SKBR3 cell line. In vitro expression of the ER/pcDNA3 plasmids and a control transfection experiment using SKBR3 cells. [file 1472-6882-13-133-S2.pdf]

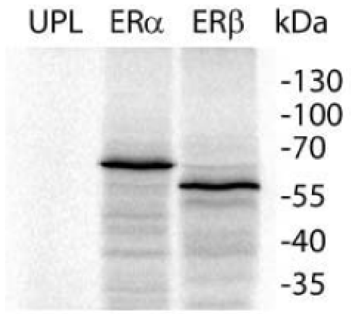

UPL, unprogrammed lysate (reaction without DNA)

1 ul of TNT reaction performed with either ERa/pcDNA3 or ERb/pcDNA3 was separated on SDS-PAGE and visualized by FLA9000 analysis (FujiFilm).

Fig 1Sa. ^{35}S *in vitro* protein expression of pcDNA3ERα (Lane 2) and pcDNA3ERβ (Lane 3) results in proteins of correct weight (66 kDa and 59 kDa for ERα and ERβ, respectively). A control lane (no DNA added) was included in Lane 1. A PageRuler prestained protein ladder (Fermentas) was included in the gel for size reference.

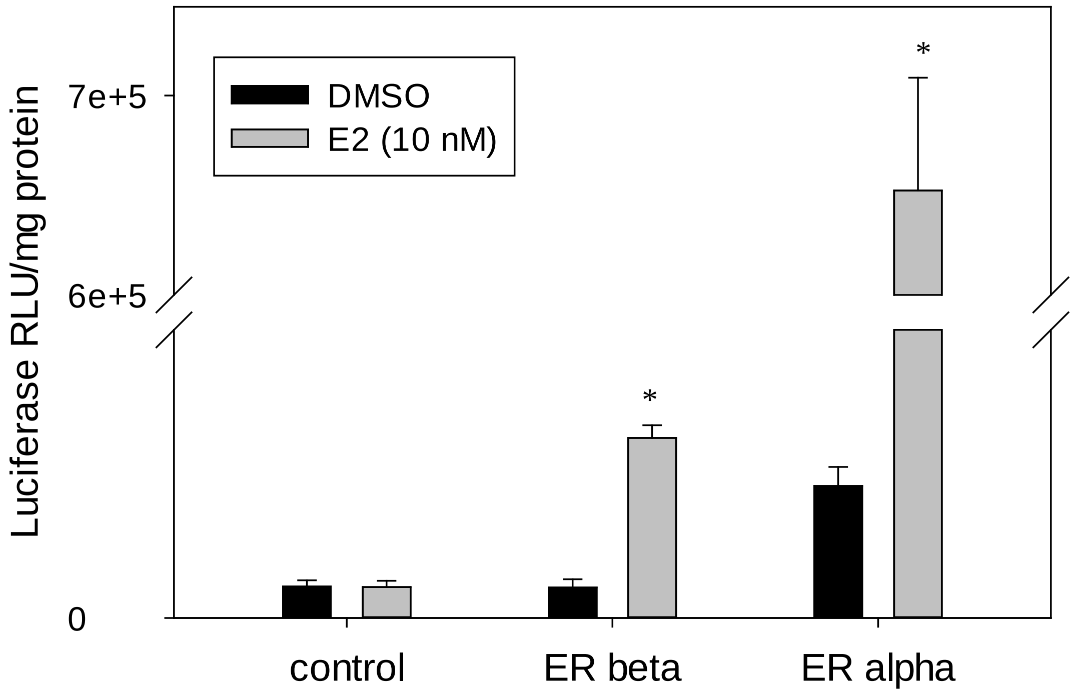

Fig 1Sb. SKBR3 cells exposed to E2 do not express luciferase if not transfected with ER. SKBR3 cells were transfected with 0.2 μg ERE-Luc plasmid, 0.05 μg of either pcDNA3ERα or pcDNA3ERβ, and 0.55 μg pcDNA3.1+. Control SKBR3 cells were transfected with 0.2 μg ERE-Luc plasmid and 0.6 μg pcDNA3.1+. Cells were treated for 24 h, lysed, and luciferase activity assessed. Luciferase expression was normalized using the Bradford assay.
