# Supplementary material for: 2,3-cis-2R,3R-(−)-epiafzelechin-3-O-p-coumarate, a novel flavan-3-ol isolated from Fallopia convolvulus seed, is an estrogen receptor agonist in human cell lines
Source: BMC Complement Altern Med. 2013 Jun 14;13:133. doi: 10.1186/1472-6882-13-133 (PMC3695784; doi:10.1186/1472-6882-13-133)
Supplement: Additional file 3 — HPLC chromatograms of F. convolvulus seed and crude fractions. HPLC chromatograms of active (estrogenic) crude fractions and whole seed extracts. [file 1472-6882-13-133-S3.pdf]

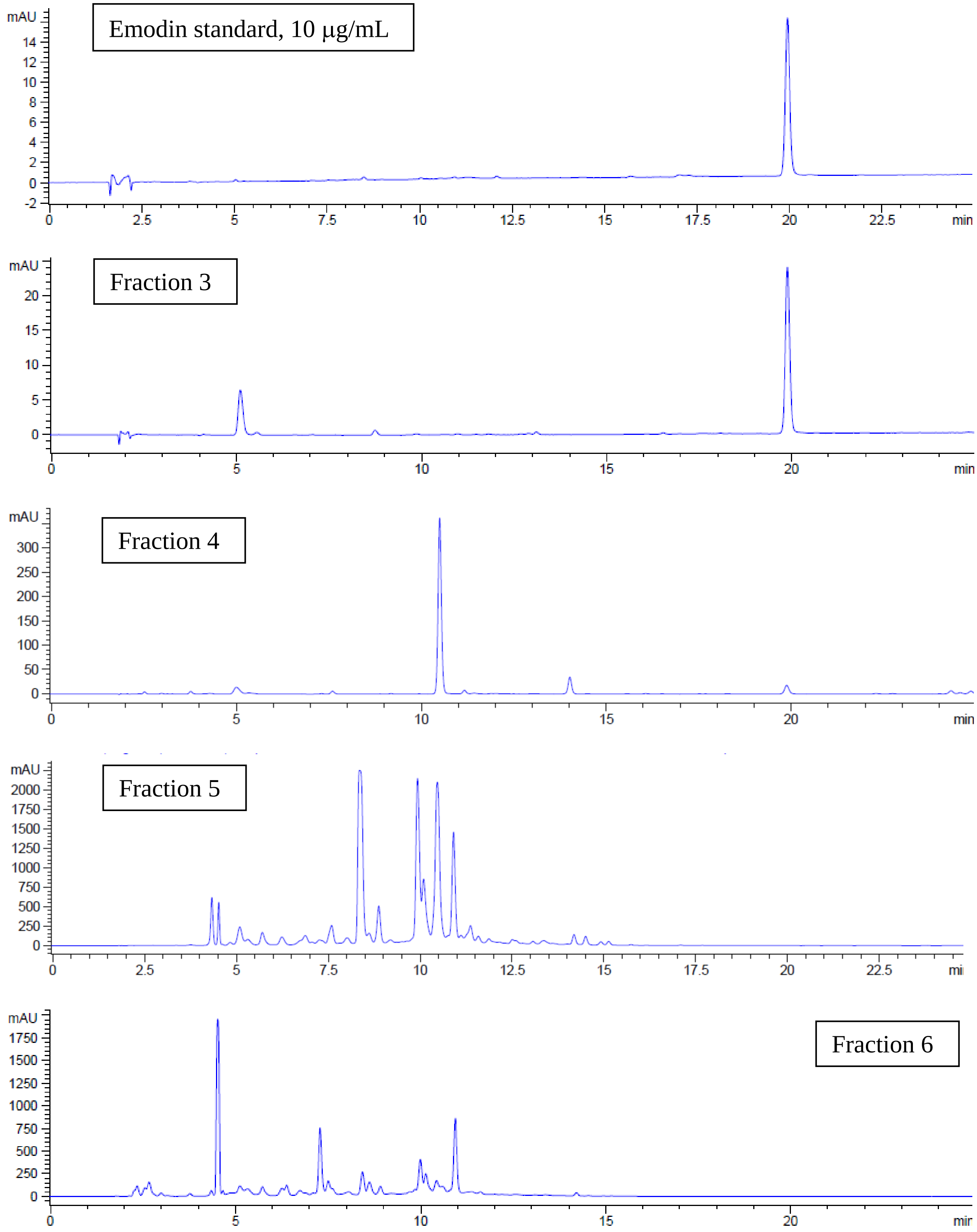

Emodin standard, 10 µg/mL
mAU
14
12
10
8
6
4
2
0
-2
0
2.5
5
7.5
10
12.5
15
17.5
20
22.5
min
Fraction 3
mAU
20
15
10
5
0
0
5
10
15
20
min
Fraction 4
mAU
300
250
200
150
100
50
0
0
5
10
15
20
min
Fraction 5
mAU
2000
1750
1500
1250
1000
750
500
250
0
0
2.5
5
7.5
10
12.5
15
17.5
20
22.5
Fraction 6
mAU
1750
1500
1250
1000
750
500
250
0
0
5
10
15
20

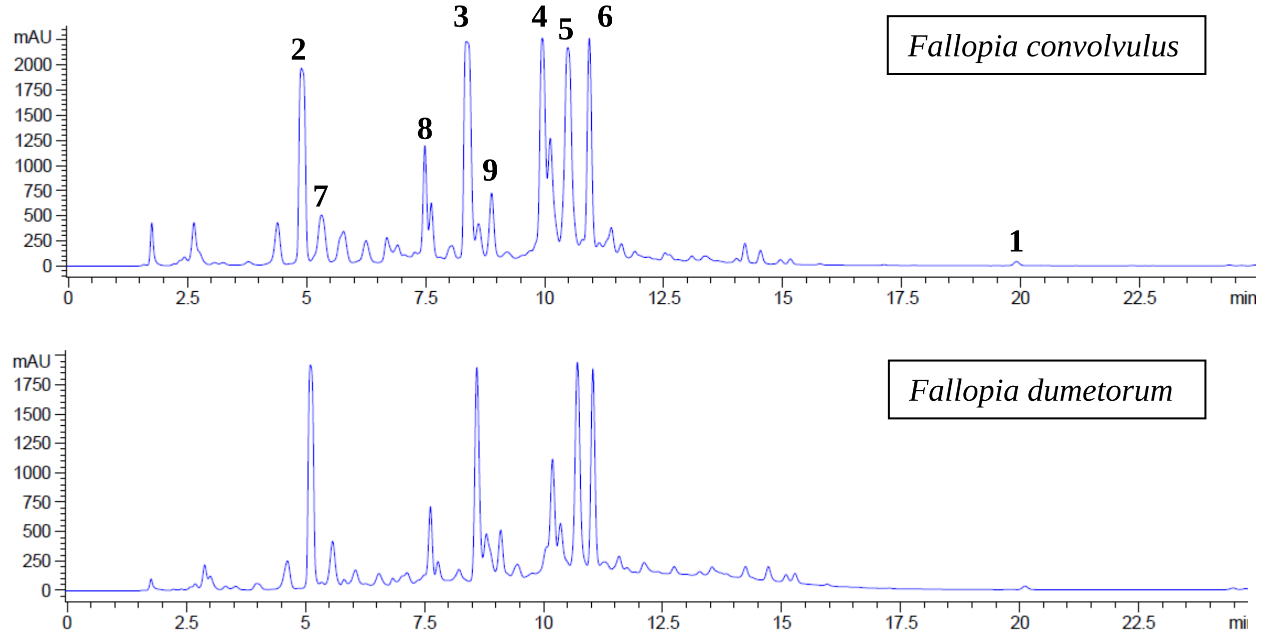

Fig. 2S. HPLC-DAD chromatograms of emodin standard (10 µg/mL), estrogenic crude fractions of *F. convolvulus* seed (8 g/mL, crude fraction 3 [20:80 EtOAc-hex separation], crude fraction 4 [50:50 EtOAc-hex separation], crude fraction 5 [EtOAc separation], crude fraction 6 [5:95 EtOH-EtOAc separation]), and whole seed extracts of *F. convolvulus* (8 g/mL) and *F. dumetorum* (8 g/mL) in EtOH. All chromatograms are at 280 nm. Polyphenolic compounds (shown here as Peaks 1-9) are discussed in Table 1S.
