# Supplementary material for: 2,3-cis-2R,3R-(−)-epiafzelechin-3-O-p-coumarate, a novel flavan-3-ol isolated from Fallopia convolvulus seed, is an estrogen receptor agonist in human cell lines
Source: BMC Complement Altern Med. 2013 Jun 14;13:133. doi: 10.1186/1472-6882-13-133 (PMC3695784; doi:10.1186/1472-6882-13-133)
Supplement: Additional file 4: Table S1 — Retention times, relative abundance, and elemental composition of pure standards and polyphenolic compounds of interest from F. convolvulus seed (shown as chomatographic peaks 1-9 which correspond to compounds 1-9). Table S2. Validation of HPLC-DAD method. Table S3. Declustering potential (DP), Ionization potential (FP), Entrance potential (EP), Collision Energy (CE), Collision cell entrance potential (CEP), Collision cell exit potential (CXP) and dwell time of selected transitions for the emodin standard. [file 1472-6882-13-133-S4.pdf]

Table 1S. Retention times, relative abundance, and elemental composition of pure standards and polyphenolic compounds of interest from *F. convolvulus* seed (shown as chomatographic peaks 1-9 which correspond to compounds 1-9).

Peak	T_R (min)	Major ions detected (negative ion) *m/z*	Respective relative abundance	λmax (nm)	Content (mg/kg)	Elemental composition	Molecular weight (g/mol)	Identification
	20.287	269.0 [M-1], 240.9, 224.9	100%, 4%, 12%	222, 267, 288		$C_{15}H_{10}O_5$*	270.05	Emodin (standard)
1	20.293	269.1, 241.1, 224.8	100%, 8%, 13%	222, 267, 288	17.9-19.6	$C_{15}H_{10}O_5$	270.05	Emodin
2	4.976	811.7 [2M-1], 405.4 [M-1], 243.3 [M-163]	9%, 88%, 100%	215, 324	389**	$C_{20}H_{22}O_9$	406.13	Undefined
3	8.554	691.3, 545.3, 419.5, 273.3	100%, 36%, 24%, 38%	235, 315	560			Undefined
4	10.157	963.5, 691.3, 543, 395.3, 271.3, 164.3, 144.9	100%, 28%, 76%, 40%, 60%, 20%, 28%	230, 315	241			Undefined
5	10.716	840.5, 419.4 [M-1], 273, 254.9, 229.1, 164.9, 145.1	8%, 77%, 100%, 16%, 11%, 27%, 50%	229, 315	278**	$C_{24}H_{20}O_7$	420.12	(−)-epiafzelechin-3-O-p-coumarate
6	10.962	581.5 [M-1], 297.4, 273, 164.9	57%, 25%, 100%, 18%	226, 285, 315	194	$C_{30}H_{30}O_{12}$	582.17	Undefined
7	5.568	817.3, 543.3, 405.3, 270.9, 243.4, 162.9, 119.2	63%, 63%, 63%, 87%, 100%, 21%, 58%	226, 310	43			Undefined
8	7.559	871.8 [2M-1], 435 [M-1], 273 [M-163], 243, 166.9	6%, 44%, 17%, 100%, 25%	224, 282	69	$C_{21}H_{24}O_{10}$	436.14	Undefined
9	8.767	713.3, 691.2, 545.3, 471, 419.6, 271.1, 145.1	22%, 100%, 30%, 33%, 37%, 44%, 29%	298	102			Undefined

*Emodin was used as external standard for calibrating accurate mass of compounds for determining elemental composition.
**Amount of compounds 2 and 5 were determined by purification and weighing. For compound 1 and compounds 3-4 and 6-9, emodin (10 µg/mL) and compound 5 abundances, respectively, at 280 nm were used as calibration for determining approximate amount of compounds (mg) in seed (1 kg).

Table 2S: Validation of HPLC-DAD method.

Compound	LOD (μg/mL)	LOQ (μg/mL)	R^2	Inter-day variation (% RSD)	Intra-day variation (% RSD)	Accuracy (% recovery)	Precision (% RSD)
Emodin	0.217	0.724	1.0000	0.43	0.46	112	13.1

Table 3S: Declustering potential (DP), Ionization potential (FP), Entrance potential (EP), Collision Energy (CE), Collision cell entrance potential (CEP), Collision cell exit potential (CXP) and dwell time of selected transitions for the emodin standard.

	m/z 269→269	m/z 269→241	m/z 269→225
DP (V)	70	70	70
FP (V)	400	400	400
EP (V)	10	10	10
CE (V)	5	35	35
CEP (V)	14	14	14
CXP (V)	15	15	15
Dwell time (msec)	200	300	200
