# Supplementary material for: 2,3-cis-2R,3R-(−)-epiafzelechin-3-O-p-coumarate, a novel flavan-3-ol isolated from Fallopia convolvulus seed, is an estrogen receptor agonist in human cell lines
Source: BMC Complement Altern Med. 2013 Jun 14;13:133. doi: 10.1186/1472-6882-13-133 (PMC3695784; doi:10.1186/1472-6882-13-133)
Supplement: Additional file 5 — Comparison of compound 1 (in F. convolvulus seed) to emodin standard. UV spectrum, mass spectrum, and MRM transitions of compound1 (emodin). [file 1472-6882-13-133-S5.pdf]

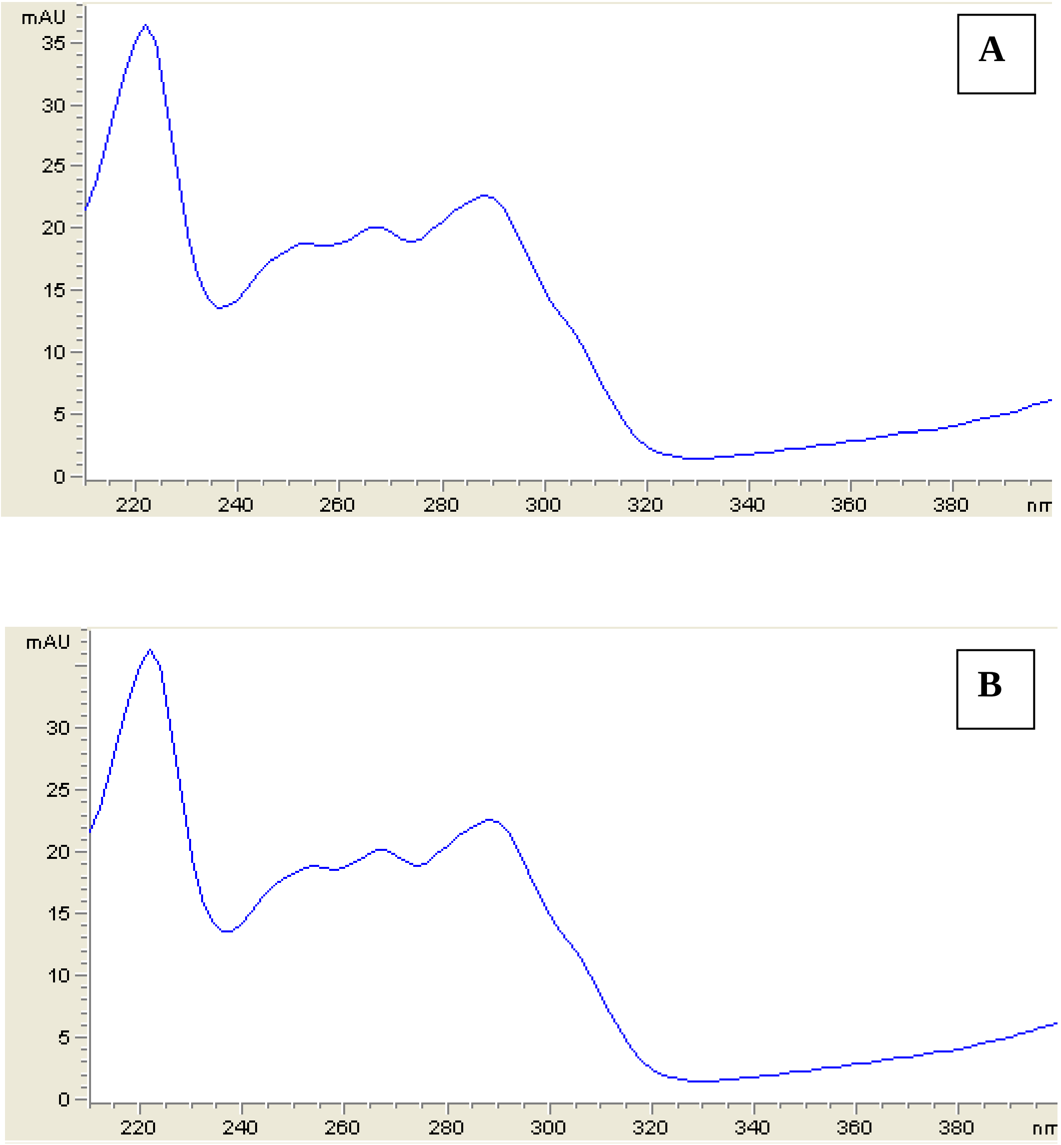

Fig. 3S. Emodin (A) and compound **1** (B) have identical UV-spectrum.

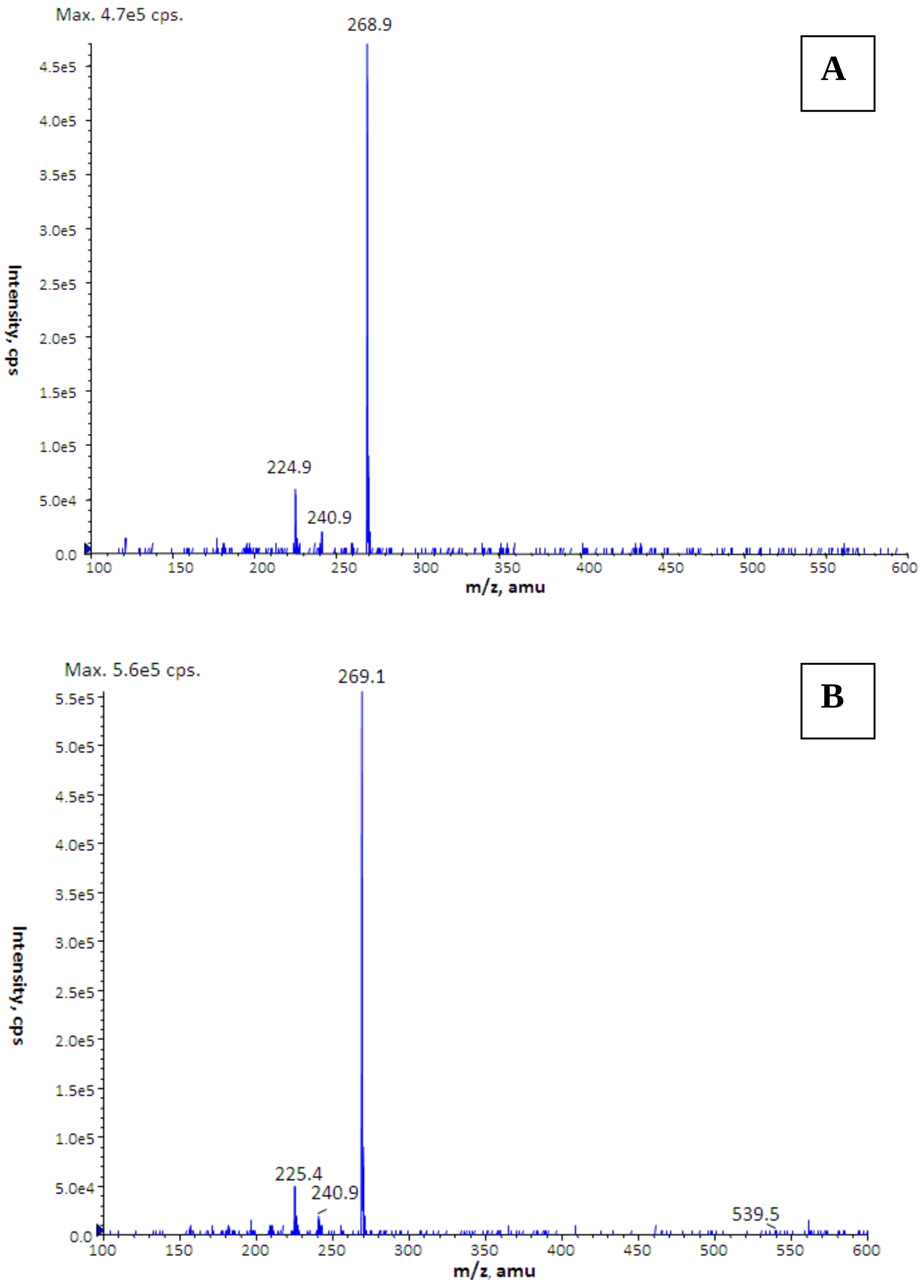

Fig 4S. Emodin (A) and compound **1** (B) contain identical peaks in their mass spectrum.

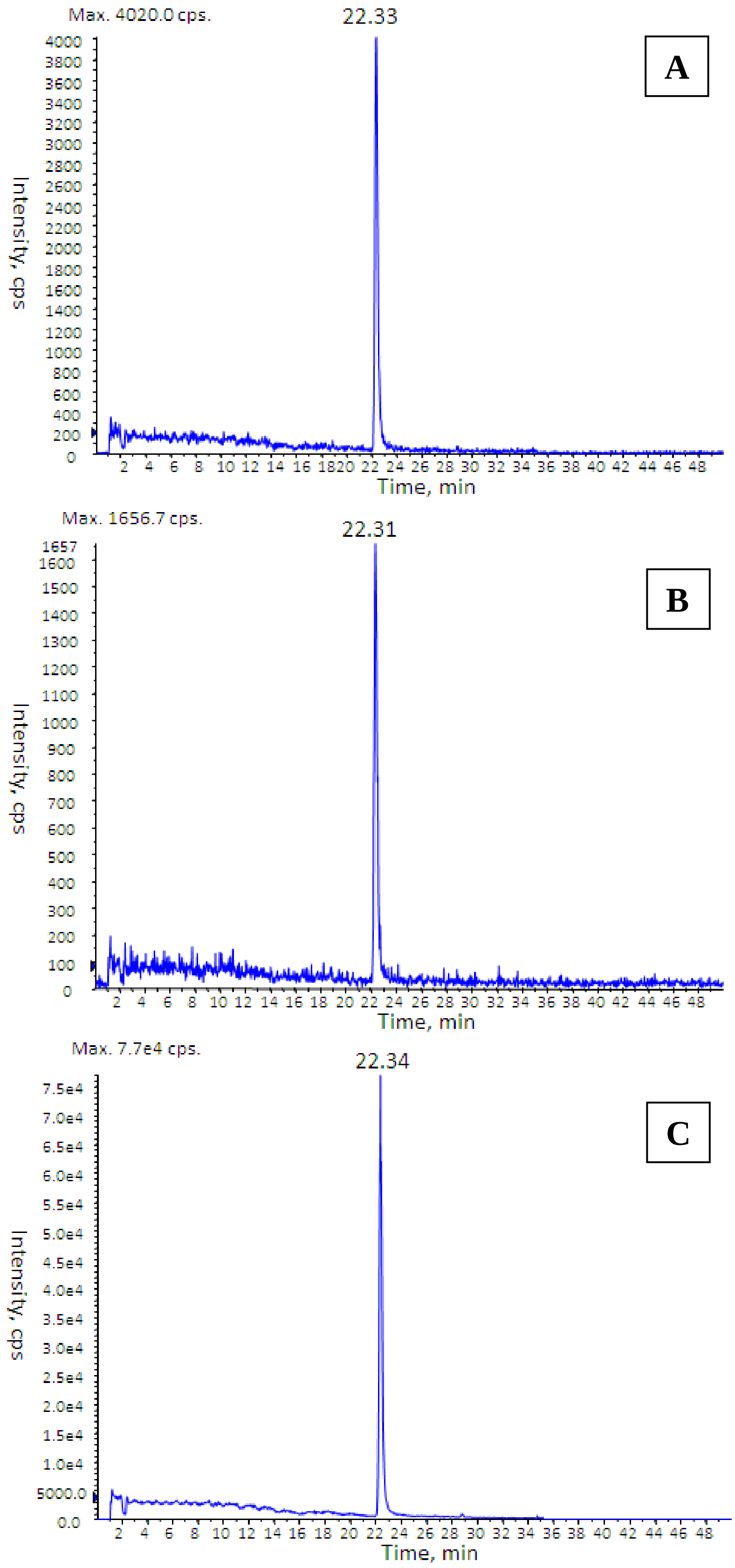
Max. 4020.0 cps.
22.33
A
Intensity, cps
Time, min
Max. 1656.7 cps.
22.31
B
Intensity, cps
Time, min
Max. 7.7e4 cps.
22.34
C
Intensity, cps
Time, min

Fig 5S. MRM transitions of compound **1** (T_R 22.3 min) match those accepted for emodin: (A) 269 m/z→225 m/z, (B) 269 m/z→241 m/z, (C) 269 m/z→269 m/z.

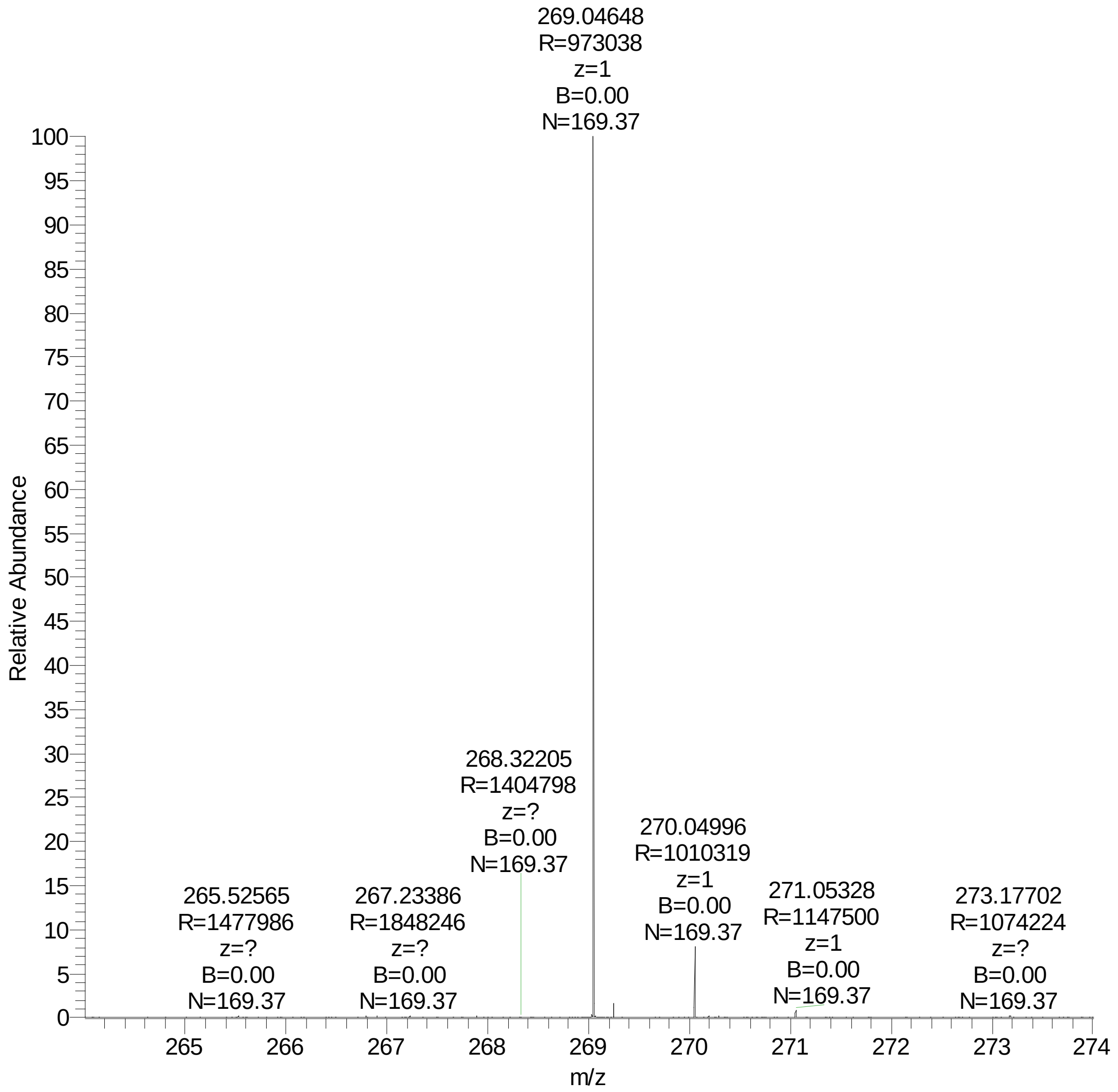

Fig. 6S. FT-ICR-MS analysis of compound **1** (emodin). Experimental accurate mass was corrected as 269.04490 using the error from the emodin standard (5.14 ppm), and elemental composition ($C_{15}H_9O_5$) was obtained from the corrected accurate mass.
