# Supplementary material for: 2,3-cis-2R,3R-(−)-epiafzelechin-3-O-p-coumarate, a novel flavan-3-ol isolated from Fallopia convolvulus seed, is an estrogen receptor agonist in human cell lines
Source: BMC Complement Altern Med. 2013 Jun 14;13:133. doi: 10.1186/1472-6882-13-133 (PMC3695784; doi:10.1186/1472-6882-13-133)
Supplement: Additional file 6 — Optimized fractionation scheme for isolation of 2,3-cis-(2R,3R)-(−)-epiafzelechin-3-O-p-coumarate from F. convolvulus seed. [file 1472-6882-13-133-S6.pdf]

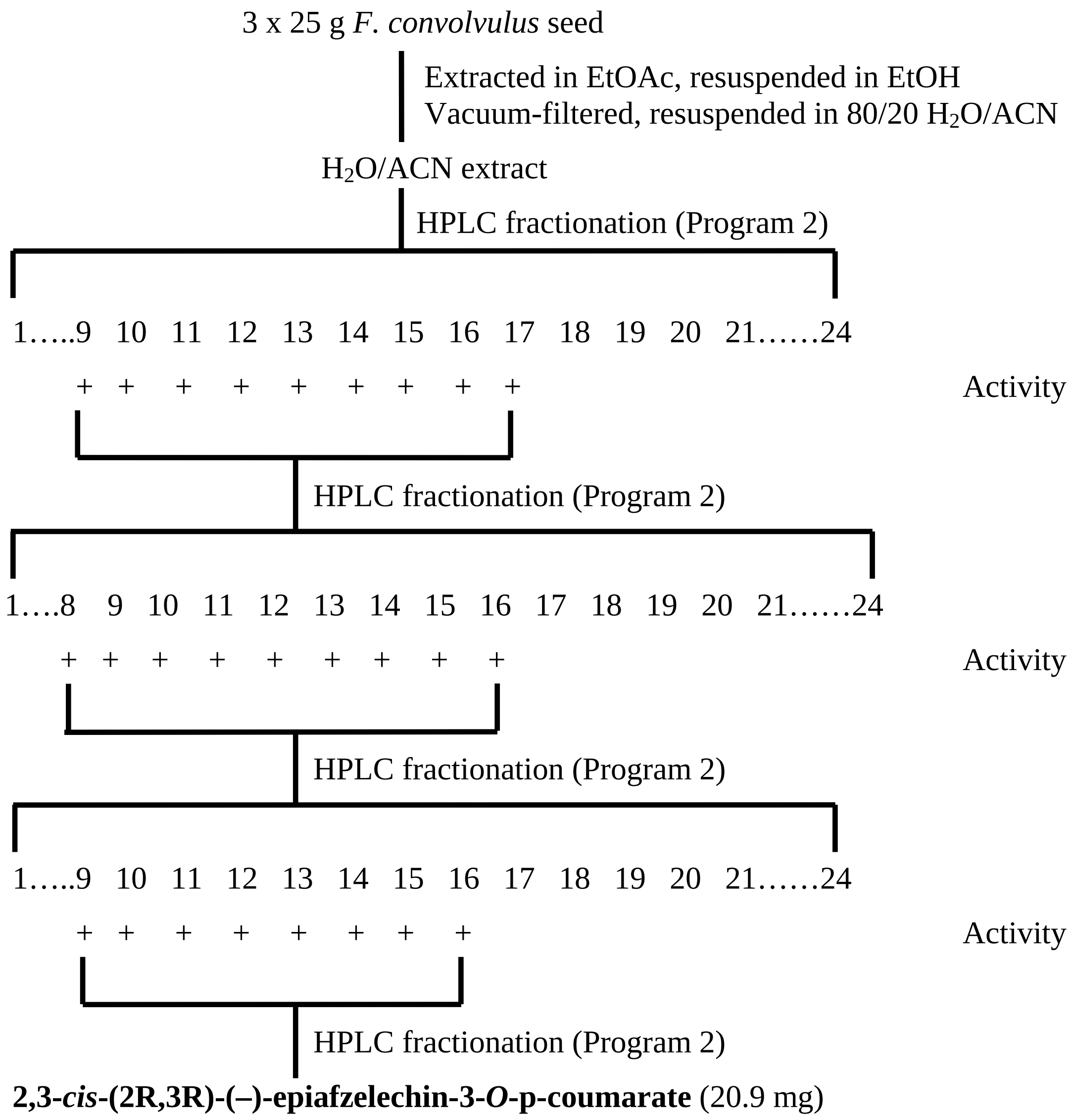

Fig 7S. Optimized fractionation scheme for isolation of 2,3-*cis*-(2R,3R)-(–)-epiafzelechin-3-*O*-p-coumarate from *F. convolvulus* seed. (+) indicates estrogenic activity in BG1Luc4E2 cell line. Crude chromatography is described in Additional file 1. HPLC programs 1 and 2 are described in Additional file 1 and Methods, respectively
