# Supplementary material for: 2,3-cis-2R,3R-(−)-epiafzelechin-3-O-p-coumarate, a novel flavan-3-ol isolated from Fallopia convolvulus seed, is an estrogen receptor agonist in human cell lines
Source: BMC Complement Altern Med. 2013 Jun 14;13:133. doi: 10.1186/1472-6882-13-133 (PMC3695784; doi:10.1186/1472-6882-13-133)
Supplement: Additional file 7 — Chemical characterization data for compound 5 (2,3-cis-(2R,3R)-(−)-epiafzelechin-3-O-p-coumarate [rhodoeosein]). UV-spectrum, mass spectra, optical rotation, and NMR spectra and correlations of rhodoeosein (isolated from F. convolvulus seed). [file 1472-6882-13-133-S7.pdf]

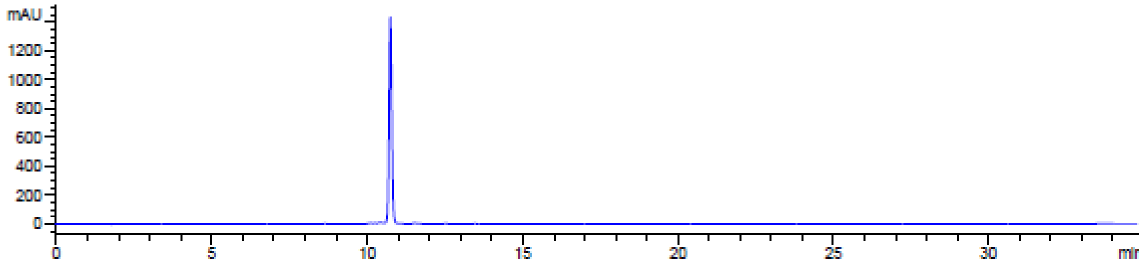

Fig 8S. HPLC-DAD chromatogram of (–)-epiafzelechin-3-*O*-p-coumarate (32 mM) at 280 nm

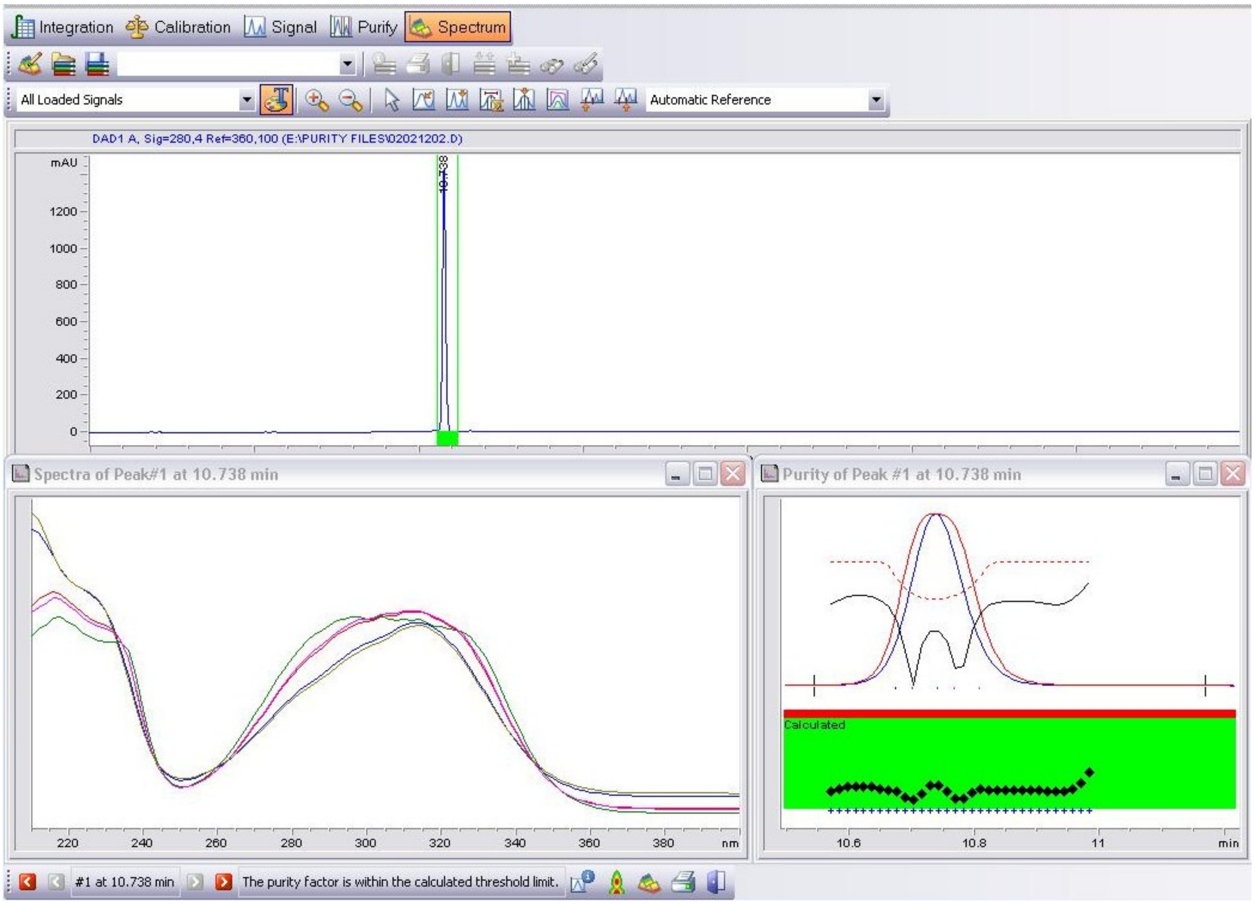

Fig 9S. Purity analysis of the peak corresponding to (–)-epiafzelechin-3-*O*-p-coumarate (32 mM) at 280 nm.

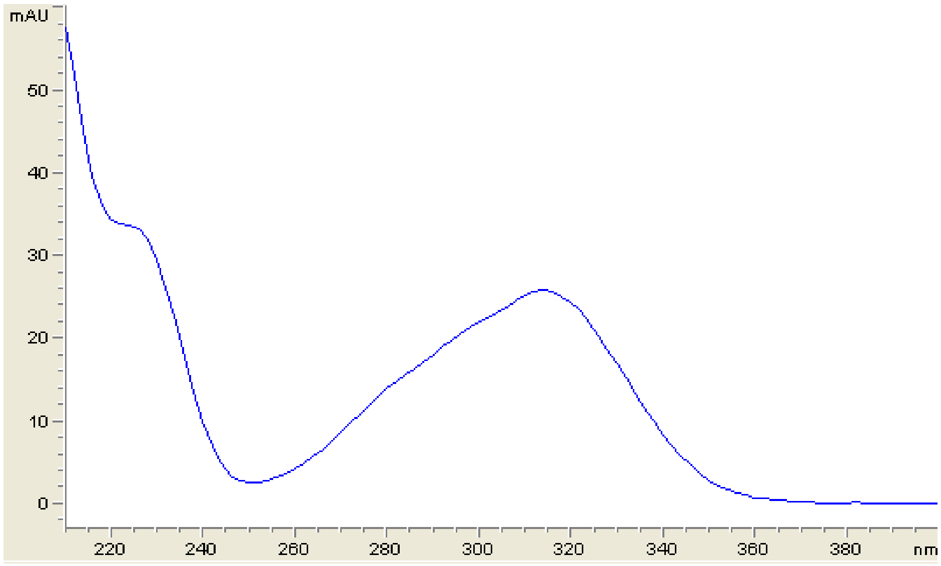

Fig. 10S. UV spectrum of (–)-epiafzelechin-3-*O*-p-coumarate (100 μM)

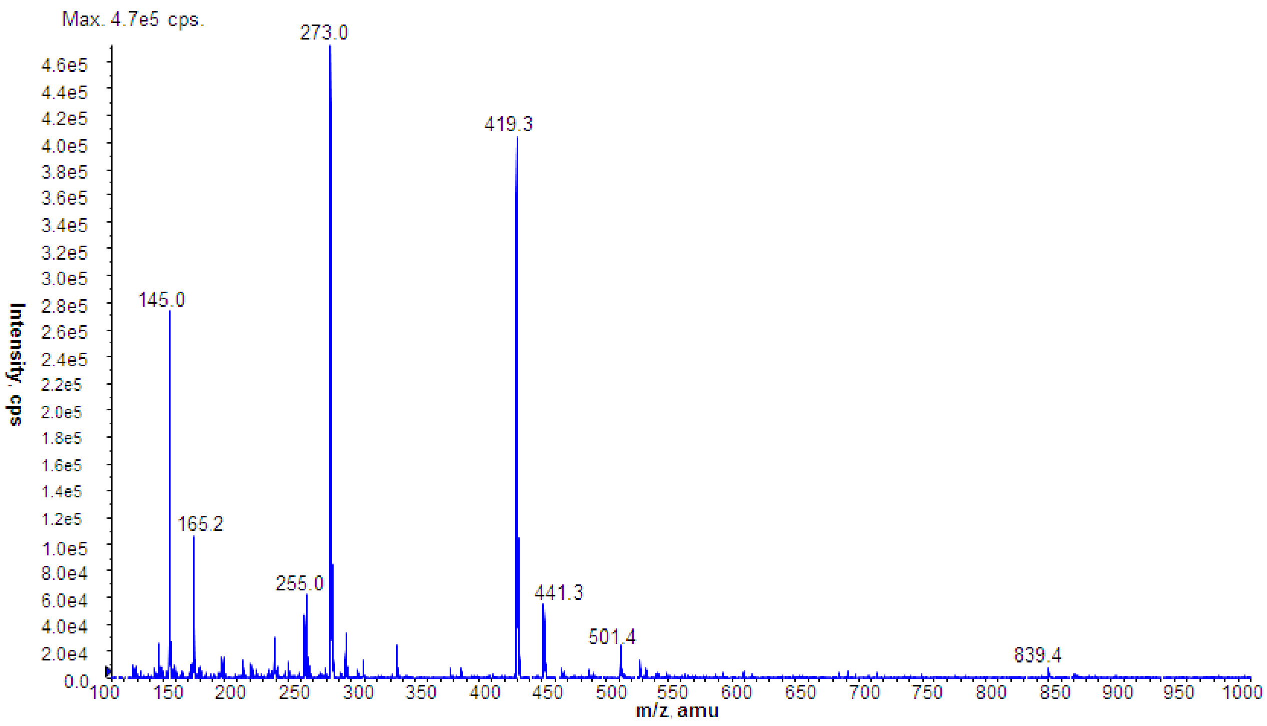

Fig. 11S. LC-(-ESI)-MS analysis of (–)-epiafzelechin-3-*O*-p-coumarate

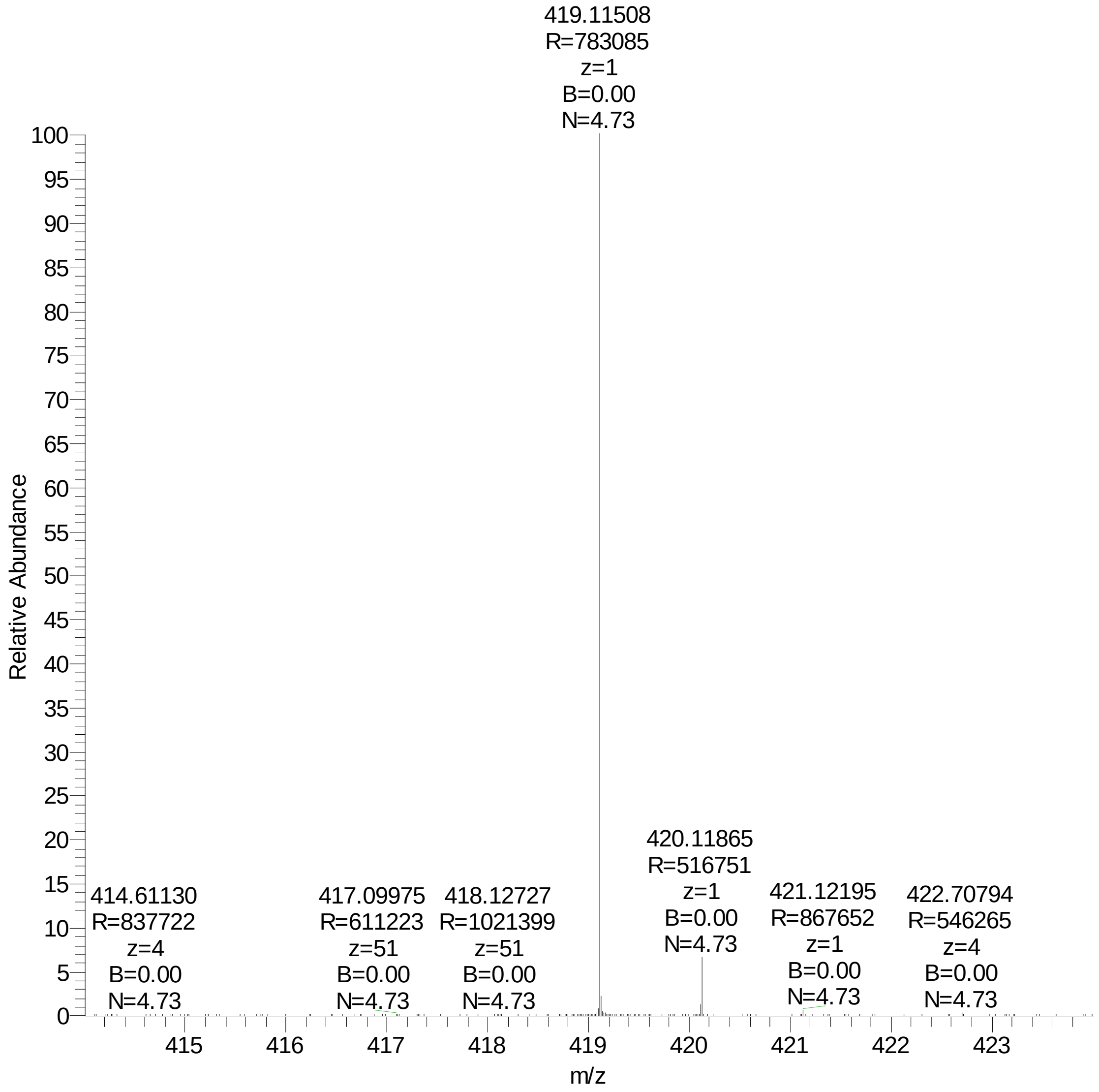

Fig. 12S. FT-ICR-MS analysis of (–)-epiafzelechin-3-*O*-p-coumarate. Experimental accurate mass was corrected as 419.11322 using the error from the emodin standard (5.14 ppm), and elemental composition ($C_{24}H_{19}O_7$) was obtained from the corrected accurate mass.

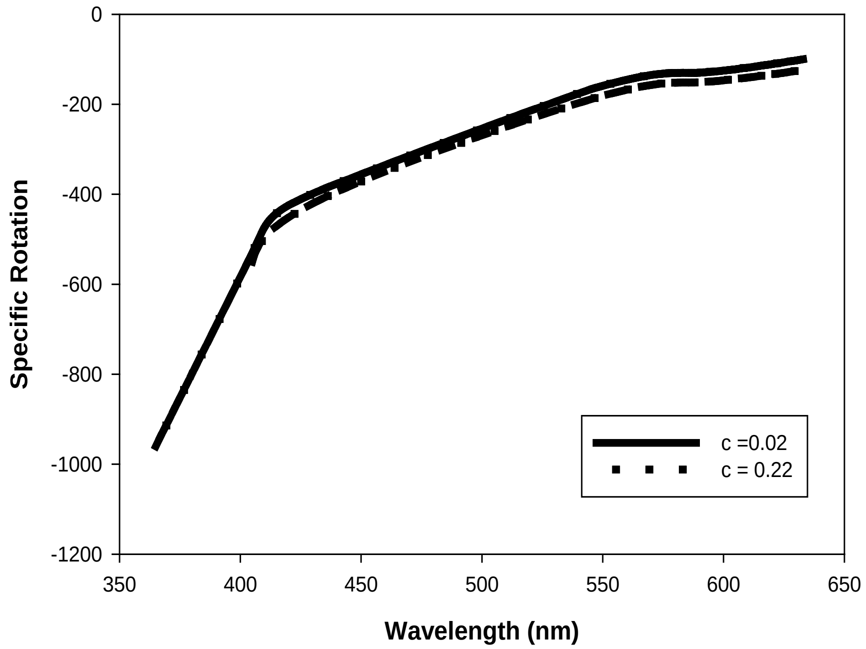

Fig. 13S. Experimental ORD spectrum of (–)-epiafzelechin-3-O-p-coumarate in MeOH solvent

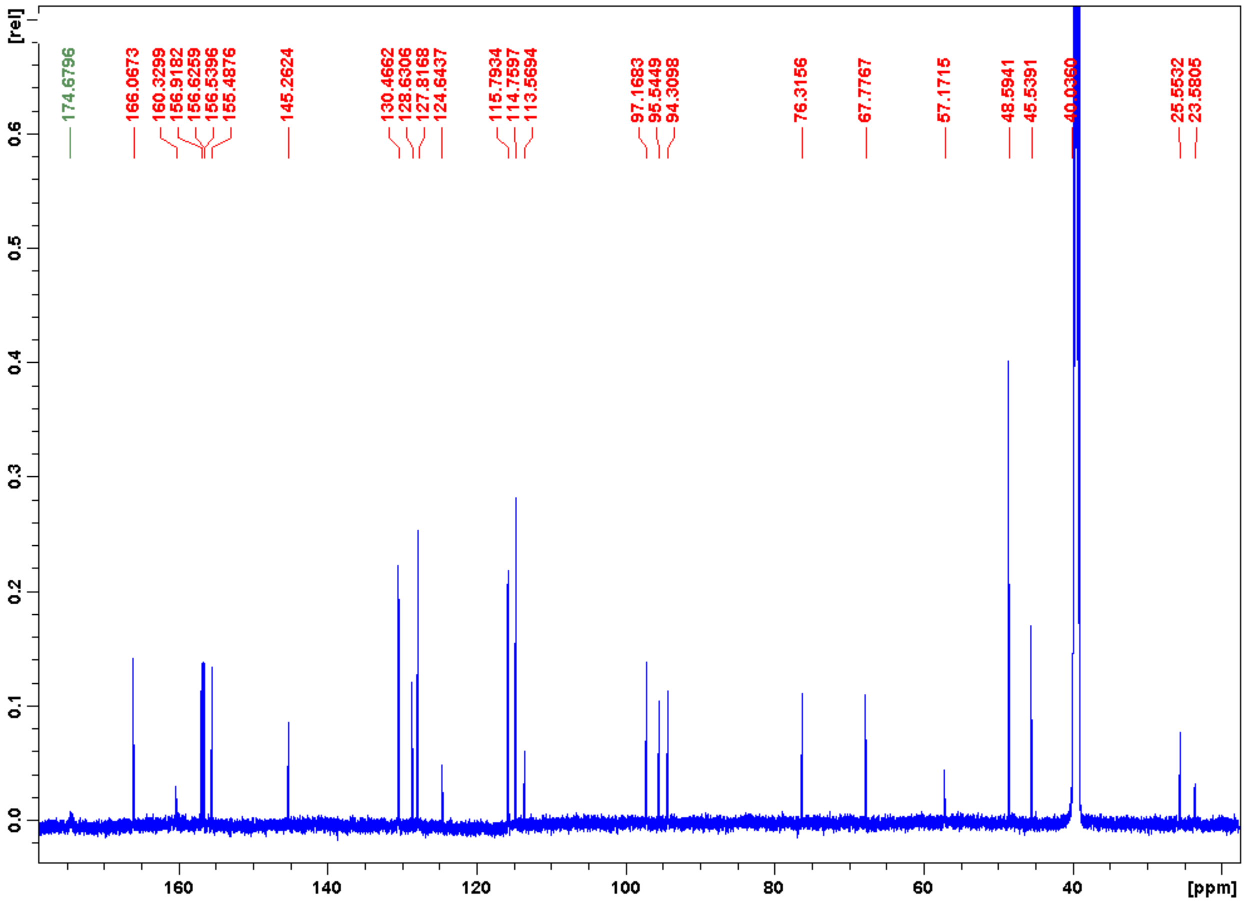

Fig. 14Sa. ^{13}C NMR spectrum of (–)-epiafzelechin-3-*O*-p-coumarate in DMSO-d6

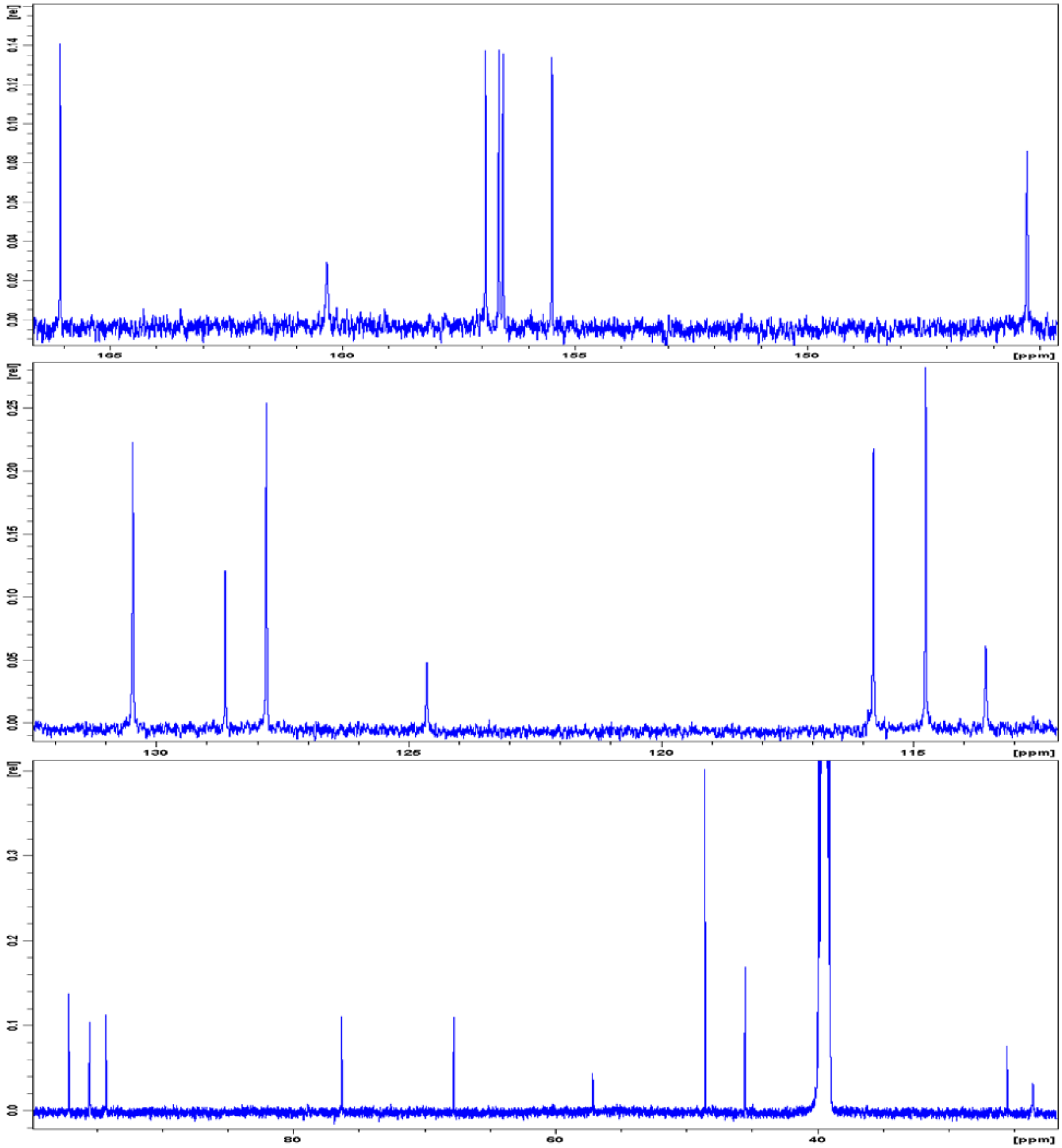

Fig. 14Sb. ^{13}C NMR spectrum (enlarged) of (–)-epiafzelechin-3-*O*-p-coumarate in DMSO-d6

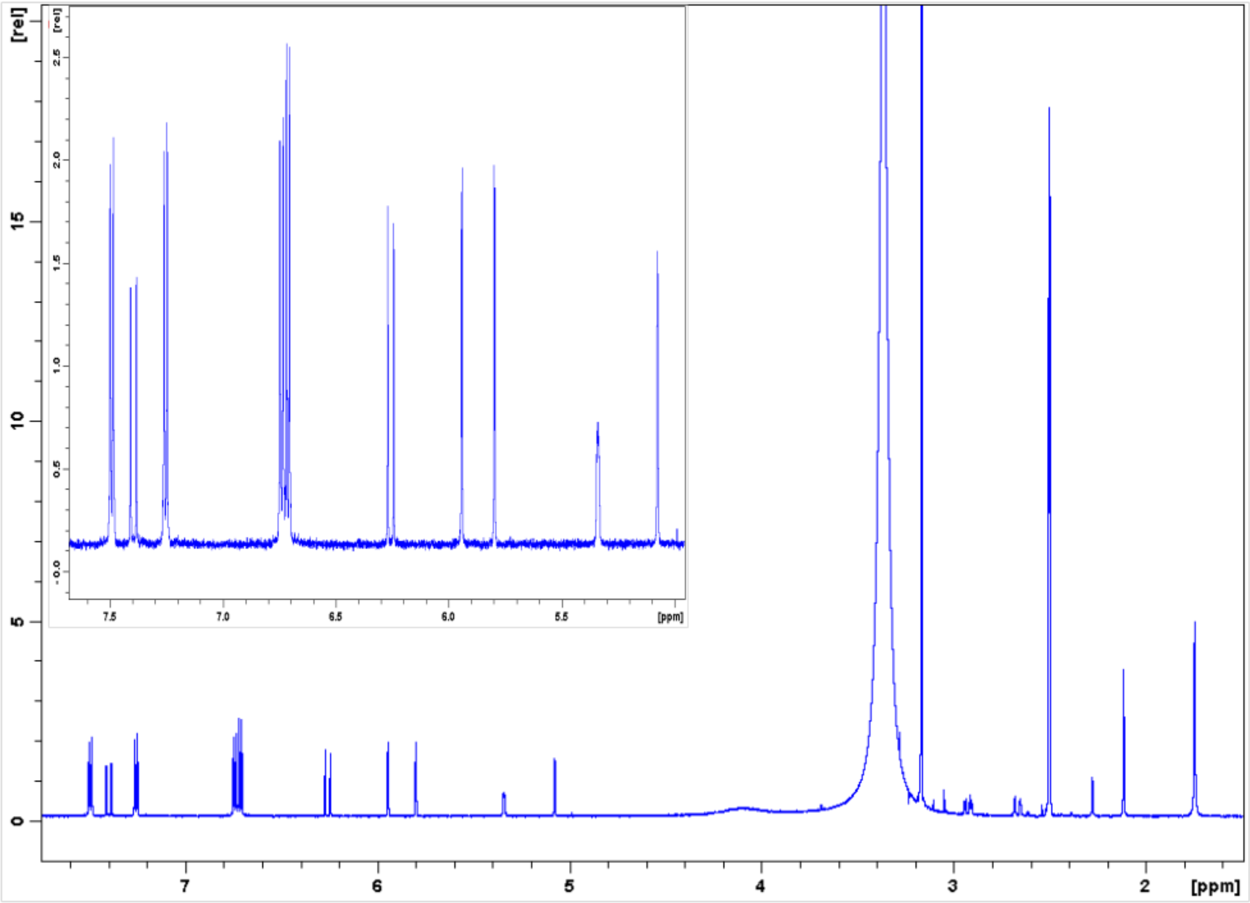

Fig. 15S. ^{1}H NMR spectrum of (–)-epiafzelechin-3-*O*-p-coumarate in DMSO-d6

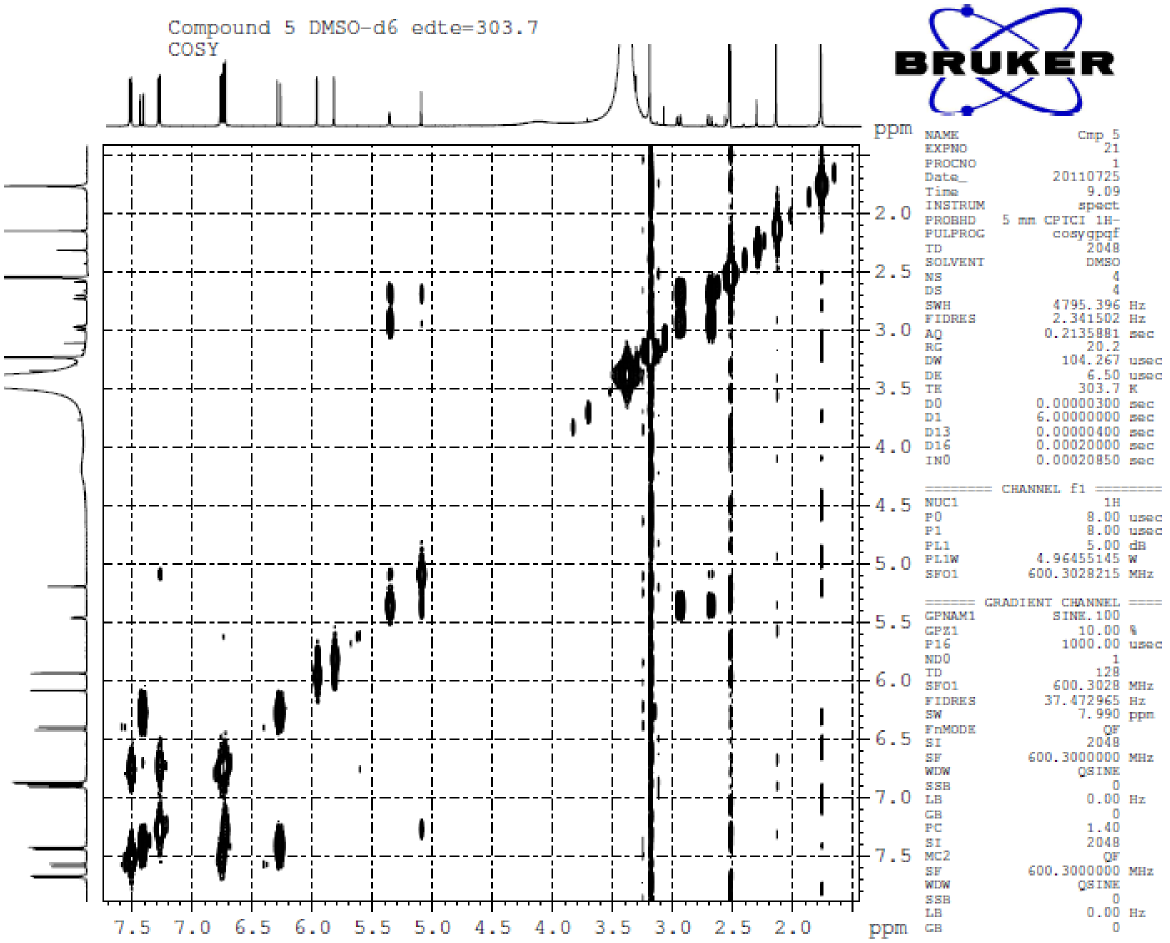

Fig. 16S. The ^{1}H-^{1}H COSY NMR spectrum of (–)-epiafzelechin-3-*O*-p-coumarate in DMSO-d6

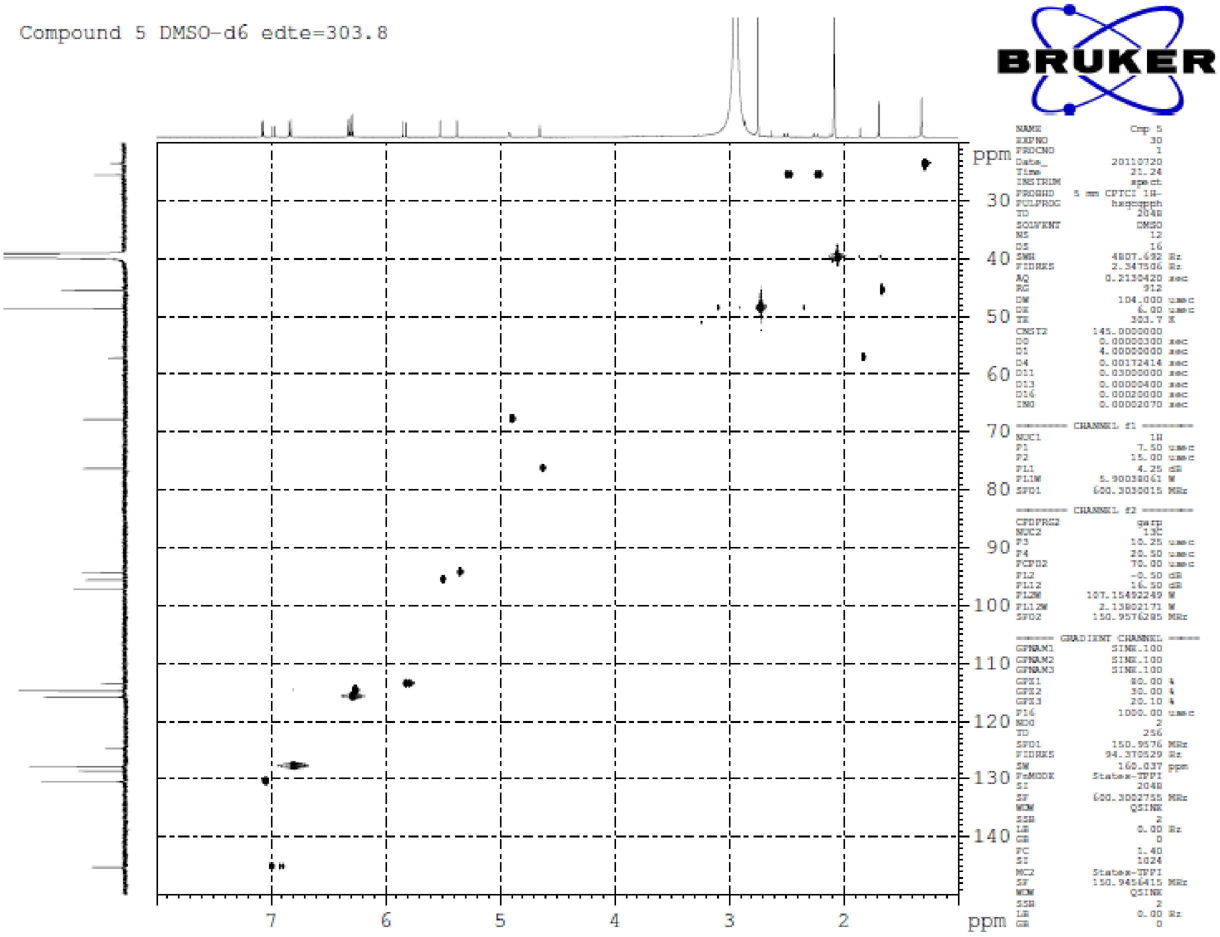

Fig. 17S. The HSQC NMR spectrum of (–)-epiafzelechin-3-*O*-p-coumarate in DMSO-d6

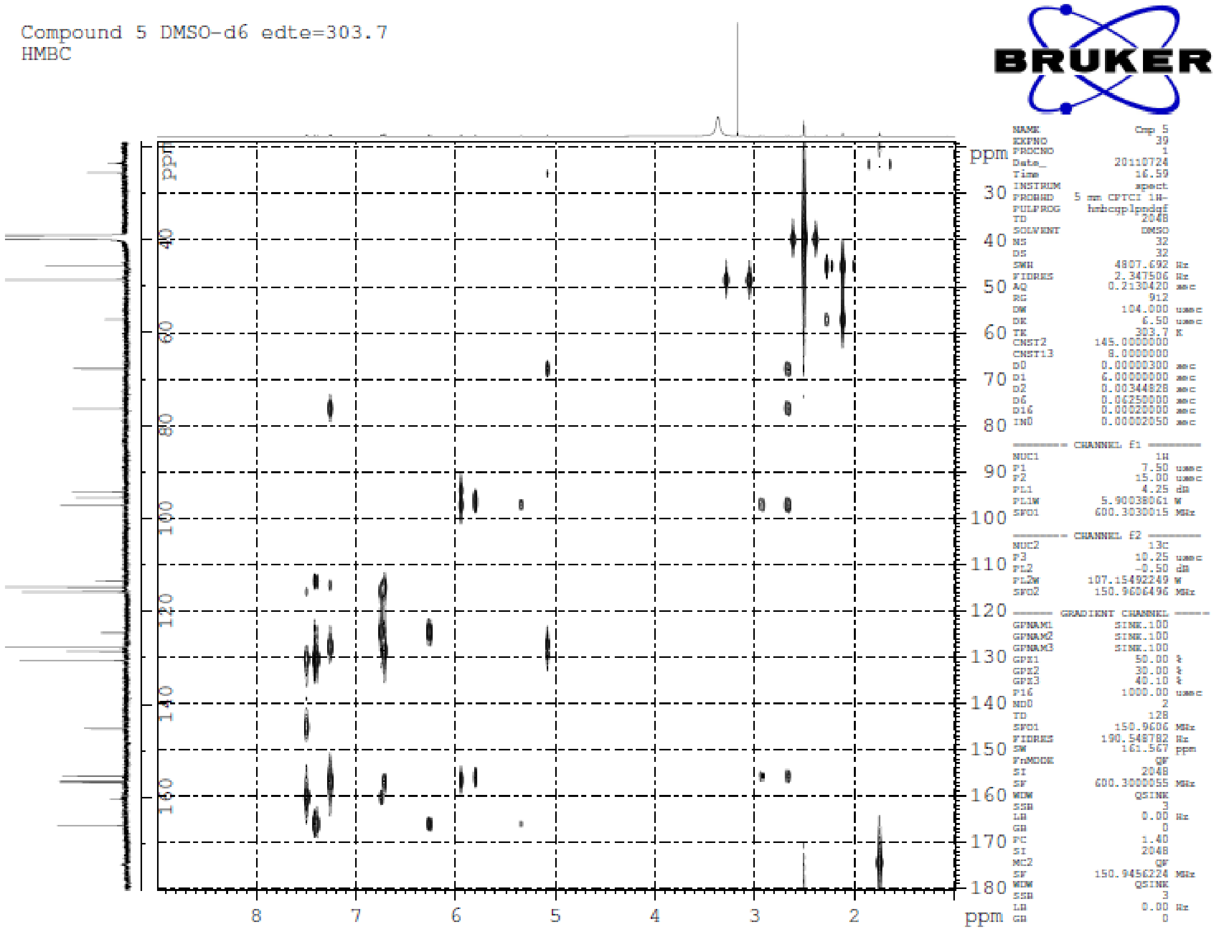

Fig. 18S. The HMBC NMR spectrum of (–)-epiafzelechin-3-*O*-p-coumarate in DMSO-d6

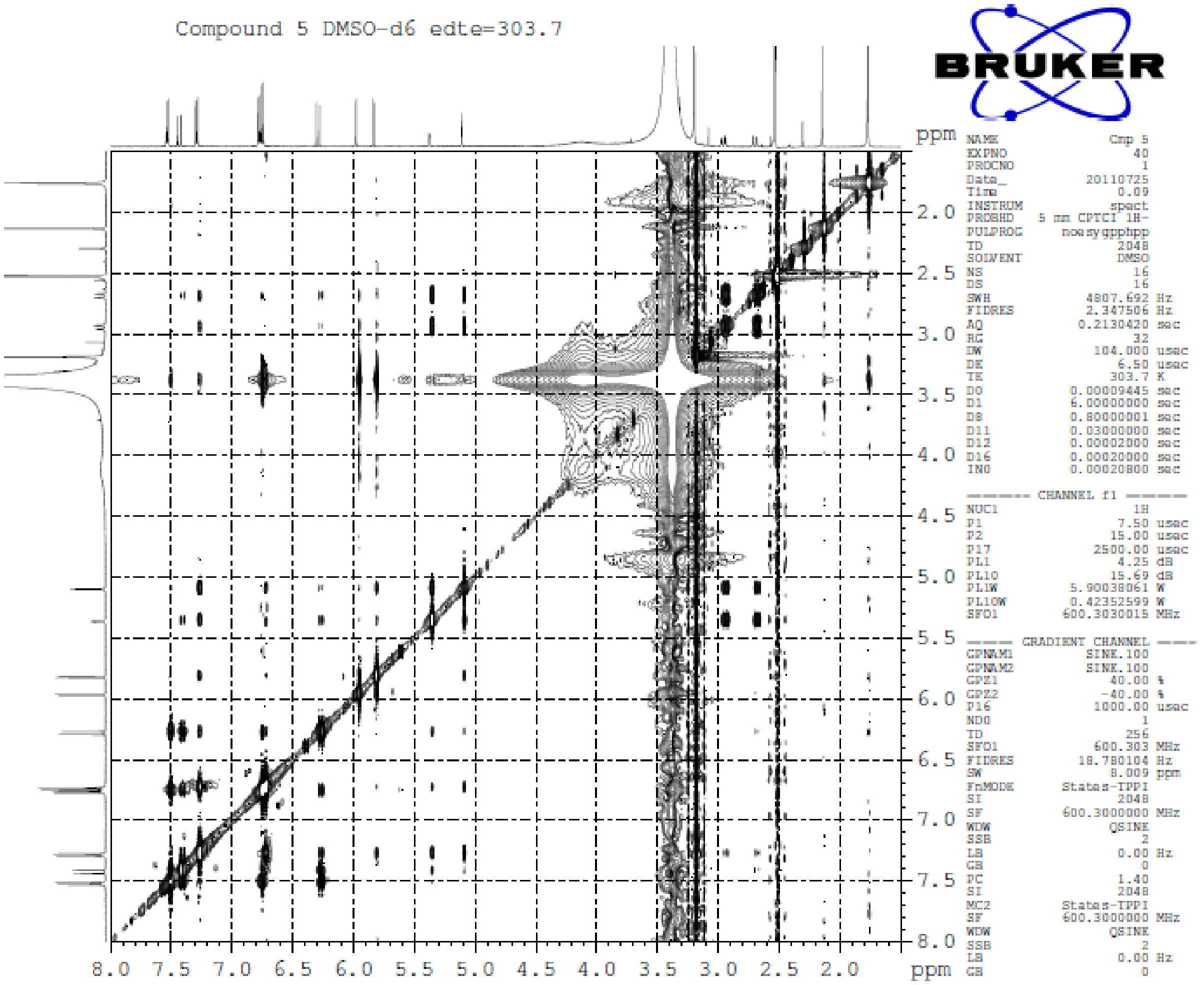

Fig. 19S. The NOESY NMR spectrum of (–)-epiafzelechin-3-*O*-p-coumarate in DMSO-d6

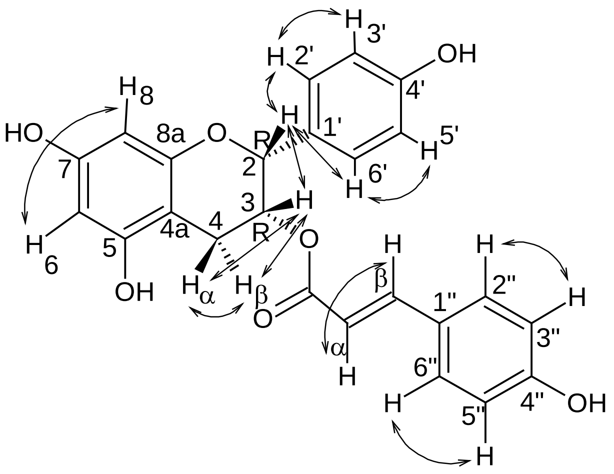

Fig. 20S. Cosy correlations were employed to identify spin-spin coupling relationships among the protons in (–)-epiafzelechin-3-*O*-p-coumarate.

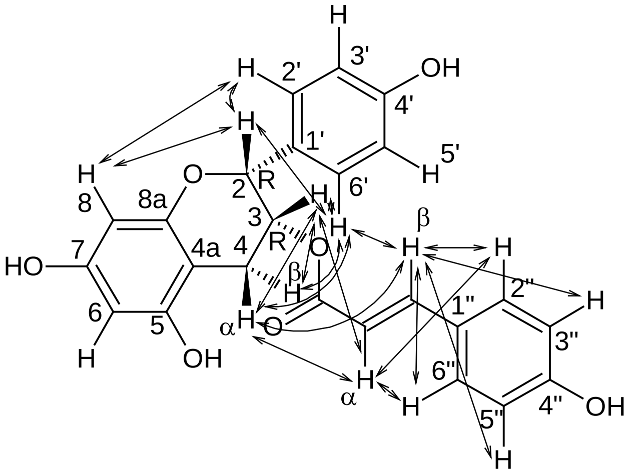

Fig. 21S. Noesy correlations determine spatial relationships between protons in (–)-epiafzelechin-3-*O*-p-coumarate.

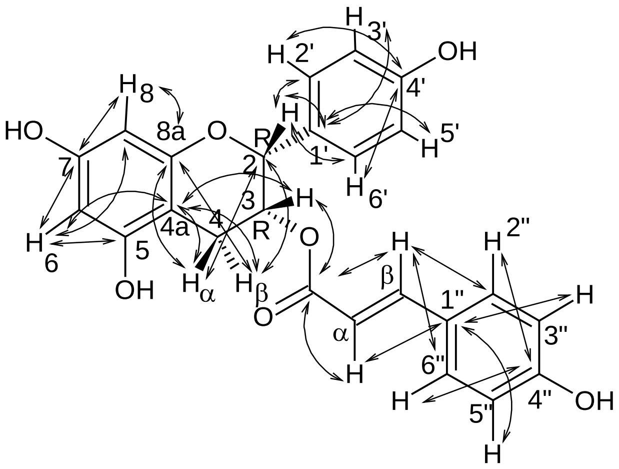

Fig. 22S. HMBC correlations identify long-range couplings between protons and carbons in (–)-epiafzelechin-3-*O*-p-coumarate.
